# Collagen VII Is Associated with Airway Remodeling, Honeycombing, and Fibroblast Foci in Usual Interstitial Pneumonia/Idiopathic Pulmonary Fibrosis

**DOI:** 10.1016/j.ajpath.2025.03.013

**Published:** 2025-04-29

**Authors:** Barbora Svobodová, Anna Löfdahl, Måns Kadefors, Salad Mohamed Ali, Oskar Rosmark, Pavan Prabhala, Mattias Magnusson, Hans Brunnström, Sofia Lundin, Göran Dellgren, Catharina Müller, Linda Elowsson, Gunilla Westergren-Thorsson

**Affiliations:** ∗ Lung Biology, Department of Experimental Medical Science, Lund University, Lund, Sweden; † Department of Clinical Chemistry and Pharmacology, Office for Medical Services, Region Skåne, Lund, Sweden; ‡ Division of Molecular Medicine and Gene Therapy, Lund Stem Cell Center, Lund University, Lund, Sweden; § Department of Pathology, Regional Laboratories Region Skåne, Lund, Sweden; ¶ Division of Pathology, Department of Clinical Sciences Lund, Lund University, Lund, Sweden; ‖ Translational Science and Experimental Medicine, Research and Early Development, Respiratory and Immunology, BioPharmaceuticals R&D, AstraZeneca, Gothenburg, Sweden; ∗∗ Department of Cardiothoracic Surgery and Transplant Institute, Sahlgrenska University Hospital, Gothenburg, Sweden

## Abstract

Collagen VII is an essential anchoring protein in the basement membrane zone, maintaining the attachment of stratified and pseudostratified epithelia to the underlying interstitial matrix. However, collagen VII is largely unexplored in normal lungs and idiopathic pulmonary fibrosis (IPF), a disease characterized by excessive accumulation of extracellular matrix and aberrant re-epithelialization of fibrotic lung parenchyma. Analysis of collagen VII protein and mRNA encoded by *COL7A1* gene in IPF distal lungs demonstrated elevated levels compared with those in normal lungs. To investigate its cellular source and spatial distribution in lung tissue, immunohistochemistry, RNAscope *in situ* hybridization, and cell culture experiments, in combination with analysis of public transcriptomic data sets were performed. In the IPF lungs, collagen VII was abundant in pathologically remodeled airways and honeycomb cysts, associated with increased basal cell populations. In contrast, in the control lungs, collagen VII was mainly localized in larger airways. RNA sequencing data revealed that epithelial basal cells and KRT5^–^/KRT17^+^ aberrant basaloid cells are the primary sources of COL7A1 mRNA expression. Furthermore, COL7A1 mRNA was observed in mesenchymal subsets, and both COL7A1 mRNA and the protein were observed in fibroblast foci, another histopathologic feature of IPF. *In vitro*, COL7A1 mRNA expression was increased in normal human lung fibroblasts treated with transforming growth factor-β1. These findings suggest that collagen VII could be involved in the process of abnormal re-epithelialization in lung fibrosis.

Idiopathic pulmonary fibrosis (IPF) is a progressive, chronic lung disease with a mean survival of 4 years after diagnosis, hallmarked by massive deposition of extracellular matrix (ECM) that leads to completely remodeled lung architecture and hindered pulmonary gas exchange.^1^^,^^2^ To date, no effective treatment exists, and the underlying cause of the disease is still unknown. However, it is hypothesized that repetitive epithelial injury and an uncontrolled wound healing process at the epithelial-mesenchymal interface drive the disease.^3^ Understanding the disease mechanisms and molecular complexity is crucial to identify potential targets for the treatment of patients with IPF.

The radiologic and histologic pattern of IPF is established as usual interstitial pneumonia (UIP). Characterized by high-resolution computed tomography, the UIP pattern typically displays bronchiectasis/bronchiolectasis (dilation of airways) and/or honeycombing.^2^ Microscopically, it is characterized by histopathologic features such as patchy dense fibrosis distorting the alveolar structure by destructive scarring, fibroblast foci (aggregates of pathologically activated fibroblasts), and/or honeycomb cysts. The expanding fibrotic tissue in patients with IPF pathologically alters the ECM of the lung, which highly influences cellular activity, favoring an IPF microenvironment.^4^ Different abnormal epithelial cell populations arise in the IPF distal lung, with similarities to cell types in large airways but also with altered transcriptomic programs affecting progenitor properties, mucus production, ECM deposition, senescence, and other functions.^5^, ^6^, ^7^, ^8^ Collagens represent the most abundant proteins of the ECM, which provide structural support for cell attachment and growth, in addition to the tensile strength of the lung tissue. Moreover, they play an important role in cell signaling in the basement membrane of epithelia.^9^, ^10^, ^11^ Increased amounts of collagen and dysregulation of various collagen types have been reported in IPF^12^, ^13^, ^14^, ^15^; however, knowledge of the localization and the function of less abundant collagen types, such as collagen VII, in lung fibrosis, is limited.

Collagen VII constitutes the main component of anchoring fibrils, which have been identified in the basement membrane zone of stratified and pseudostratified epithelia of several normal tissues, but not beneath simple epithelia and endothelia of blood vessels.^16^^,^^17^ In contrast, collagen IV and laminin are present in all basement membranes, including those in blood vessels.^17^ Collagen VII is composed of three identical collagen α-1 chains, each encoded by the *COL7A1* gene and expressed by both skin epithelial cells and fibroblasts in mice.^18^ In the form of anchoring fibrils, collagen VII strengthens the epithelial-stromal attachment via binding to interstitial collagen I and/or to basement membrane components collagen IV and β3 and γ2 chains of laminin 332.^19^^,^^20^ Laminin 332 together with transmembrane collagen XVII are further linked to keratin intermediate filaments 5 (KRT5) and 14 through the hemidesmosomal components integrin α6β4, plectin (PLEC), tetraspanin (CD151), and dystonin (DST).^21^ These molecules jointly form an anchoring complex of the epithelial cells (Figure 1). Interestingly, *Col7a1* null (−/−) mice die within 2 weeks after birth, and the absence of collagen VII in human skin caused by loss-of-function mutations in the *COL7A1* gene or circulating autoantibodies against collagen VII epitopes has been implicated in various skin diseases, leading to severe manifestations, such as skin blistering, finger and tooth abnormalities, skin and mucosal fibrosis, and ultimately cutaneous squamous cell carcinoma.^22^, ^23^, ^24^ Furthermore, mutations in other components of the hemidesmosomal and anchoring complexes manifest with similar severe outcomes as mutations in collagen VII in the skin disease epidermolysis bullosa,^25^ highlighting the importance of the entire structure in the basement membrane zone. Nevertheless, the role of collagen VII in lung homeostasis, fibrosis, and re-epithelialization remains unknown.

**Figure 1:**
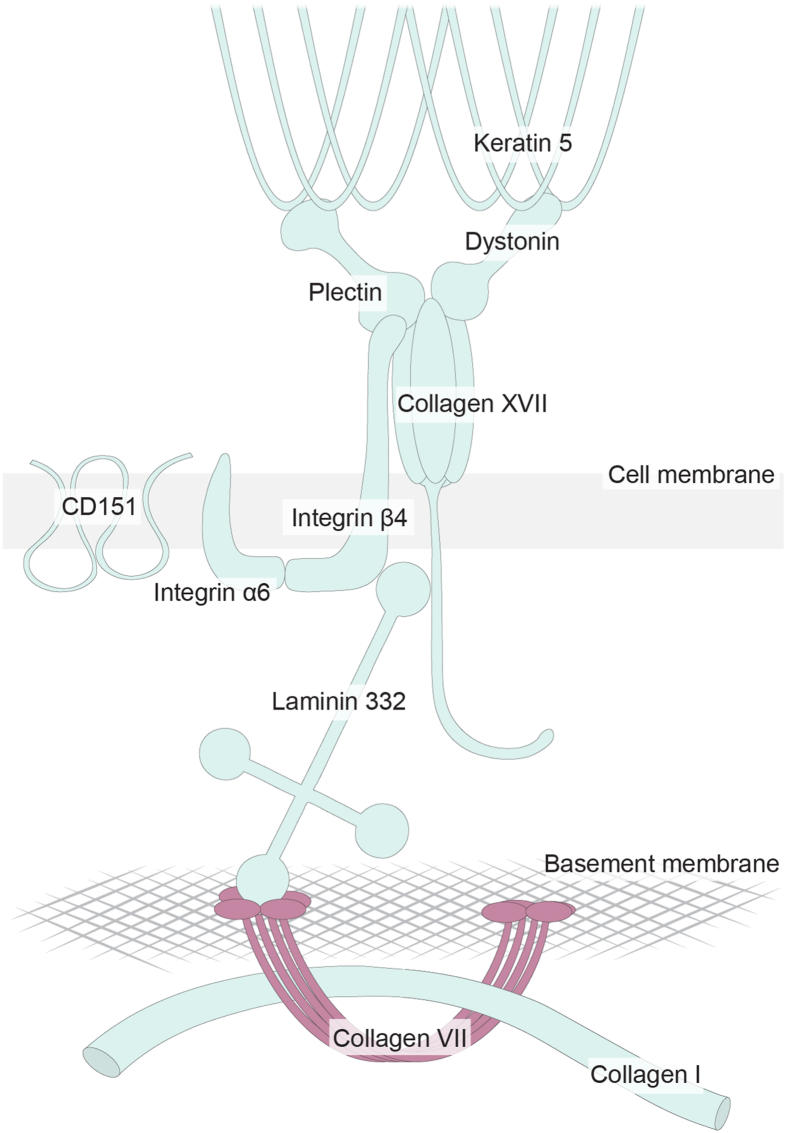
Schematic illustration of the hemidesmosomal and the anchoring complex of epithelial cells in their basement membrane zone, based on studies in skin. Illustration made by Lisa Karlsson (Lung Biology Group, Lund University, Lund, Sweden).

In conclusion, collagen VII is elevated in IPF lungs compared with control lungs and was expressed by aberrant epithelial and mesenchymal cells in remodeled IPF lung tissue, demonstrating collagen VII as a potential novel biomarker for IPF and a possible therapeutic target that would be interesting to further validate in larger studies.

## Materials and Methods

### Human Material and Study Approval

Explanted lungs from organ donors that were not suitable for lung transplantation and from patients with end-stage IPF undergoing lung transplantation were donated for research purposes and acquired in collaboration with Sahlgrenska University Hospital (Gothenburg, Sweden). The study was approved by the Swedish Research Ethical Committee in Gothenburg (Diary number 2008/413, 1026-15). In all cases, written informed consent was obtained. IPF was diagnosed by clinicians at Sahlgrenska Hospital according to the American Thoracic Society/European Respiratory Society/Japanese Respiratory Society/Asociación Latinoamericana de Tórax guidelines^26^ before lung transplantation. Organ donors had no history of lung disease, and only microscopically normal lung tissue was used as non-IPF control tissue (controls). The characteristics of both controls and patients with IPF used throughout the study are summarized in Table 1 and with details in Supplemental Table S1.

**Table 1:** Characteristics of Controls and Patients with IPF

Group	Controls	Patients with IPF
Sex, males/females	7/1	5/3
Age, means ± SD, years	60 ± 12	60 ± 5
Smoking status, never/former/current smokers	4/3/1	2/6/0

IPF, idiopathic pulmonary fibrosis.

### Mass Spectrometry Data Analysis

The proteomic data set with the identifier PXD012322, published in a previous study^4^ and deposited to the ProteomXchange Consortium (*https://proteomecentral.proteomexchange.org*, last accessed May 2, 2022) via the PRIDE partner repository,^27^ was used to identify collagen VII in control and end-stage IPF acellular distal lung tissue. To achieve better coverage of collagen-derived peptides, a new search was performed in Maxquant^28^ with the addition of N-terminal acetylation and hydroxyproline as variable modifications for peptide identification and quantification in addition to the peptide modifications used in the original publication [ie, methionine oxidation (variable) and carbamidomethylation (fixed)]. The search was performed using an updated version of Maxquant version 2.0.1.0, with the match between runs feature enabled to further improve peptide peak identification. Label-free quantification^29^ was performed to normalize peptide intensities between samples. Only values from the time point 24 hours are shown in the final graph to minimize an effect of potential ECM degradation by cells in later time points.

### Analysis of Bulk RNA-Sequencing Data

Bulk RNA-sequencing data set GSE134692^30^ from control and IPF lungs was downloaded from the Gene Expression Omnibus repository (*https://www.ncbi.nlm.nih.gov/geo*, last accessed September 3, 2022), and trimmed mean of M-values of COL7A1 was extracted. Only values from normal and IPF tissue were of interest, and to exclude potential batch effects, only samples from batch 1 were used.

### Analysis of Single-Cell RNA-Sequencing Data

Single-cell RNA-sequencing data GSE135893^8^ from 10 control lungs and 20 pulmonary fibrosis lungs [12 IPF and 8 non-IPF interstitial lung disease (ILD)] were downloaded from the Gene Expression Omnibus repository (*https://www.ncbi.nlm.nih.gov/geo*, last accessed October 12, 2023). Aligned and annotated data (GSE135893_ILD_annotated_fullsize.rds.gz) were analyzed in R version 4.3.2 (*https://www.r-project.org*) using the Seurat package version 5.0.2 (*https://cran.r-project.org/web/packages/Seurat/index.html*). Cells from patients with ILDs other than IPF were excluded, leaving a total of 89,326 cells from 10 controls and 12 IPF samples for further analysis. Cell type annotations at the finest resolution from the original study were used for all cells except for mesenchymal cells, which were annotated on the basis of consensus cell type identities established in the Human Lung Cell Atlas^31^ (Supplemental Figure S1) using the Azimuth reference-based mapping pipeline (Human–Lung v2, *https://azimuth.hubmapconsortium.org*, last accessed October 19, 2023).^32^ The reference mapping of the original mesenchymal fraction annotated all submitted cells as belonging to stroma/mesenchymal identities on all levels of annotation except for one cell, which was excluded from the analysis. The finest annotation level (ann_finest_level) from the Human Lung Cell Atlas reference was used for all mesenchymal cells except for Smooth muscle FAM83D+ and SM activated stress response, which were manually annotated as Smooth muscle. Normalization of counts (RNA data slot) using the NormalizeData function and default settings was performed before plotting data.

### Histology and Immunohistochemistry

Tissue samples from distal regions (parenchymal tissue adjacent to visceral pleura) and proximal regions (bronchi) of explanted lungs were fixed in 10% formalin, dehydrated, and embedded in paraffin [formalin-fixed, paraffin-embedded (FFPE)]. Samples were derived from controls (*n* = 6 for distal region, 2 FFPE blocks/individual; *n* = 4 for proximal region, 1 FFPE block/individual) and patients with IPF (*n* = 6 for distal region, 2 FFPE blocks/individual; *n* = 4 for proximal region, 1 FFPE block/individual). Tissue sections (4 μm thick) placed on Superfrost Plus Menzel Gläser slides (catalog number 631-9483; VWR, Radnor, PA) were incubated at 60°C for 30 to 60 minutes, followed by standard deparaffinization and rehydration. Serial tissue sections were stained either with hematoxylin and eosin (H&E) and modified Russell-Movat pentachrome (further as pentachrome) or by immunohistochemistry (further as IHC, both chromogenic and fluorescent). For chromogenic IHC, enzymatic antigen retrieval was performed with Digest-All 3 pepsin solution (catalog number 003009; Thermo Fisher Scientific, Waltham, MA) at 37°C for 20 minutes, followed by blocking with BLOXALL Endogenous Peroxidase and Alkaline Phosphatase Blocking Solution (SP-6000-100; Vector Laboratories, Newark, CA) and 2.5% Normal Horse Serum (catalog number 30022; Vector Laboratories). Rabbit polyclonal monospecific antibody recognizing the central noncollagenous-1 domain of human collagen VII (a generous gift from Alexander Nyström, Department of Dermatology, University of Freiburg, Freiburg, Germany)^33^ was diluted 1:20,000 in 2% bovine serum albumin in tris-buffered saline and incubated overnight at room temperature. The following day, tissue sections were incubated with the horseradish peroxidase–coupled secondary antibody ImmPRESS (Peroxidase) Polymer Anti-Rabbit IgG Reagent (catalog number MP-7401; Vector Laboratories), detected with ImmPACT DAV EqV Peroxidase (HRP) Substrate (catalog number SK-4103; Vector Laboratories), dehydrated and counterstained with Mayer hematoxylin to visualize nuclei. Slides were mounted with Pertex mounting medium (catalog number 00840-05; Histolab, Askim, Sweden) and a number 1 thickness cover glass. A tissue section of human normal skin was used as a positive control for rabbit polyclonal monospecific anti-collagen VII antibody, and lung tissue sections omitting the primary antibody were used as negative controls in all stainings (Supplemental Figure S2).

For fluorescent IHC, distal lung tissue sections (1 FFPE block/individual, *n* = 6 for both groups) and the same enzymatic antigen retrieval method as above were used for antibodies against collagen VII, collagen IV, pan-cytokeratin, and keratin 17 (KRT17), or heat-induced antigen retrieval method using Tris/EDTA buffer (pH 9.0) in the PT Tissue Link system (Dako, Agilent Technologies, Santa Clara, CA) for antibodies against KRT17 and KRT5, all followed by incubation with 10% goat serum for 20 minutes. Mouse monoclonal anti–pan-cytokeratin [1:1000; clone AE1/AE3; catalog number ab27988; Abcam Limited, Cambridge, UK; Research Resource Identifier (RRID): AB_777047], mouse monoclonal anti–collagen VII (1:100; clone LH7:2; catalog number ab6312; Abcam Limited; RRID: AB_305415), rabbit polyclonal anti–collagen IV (1:1000; catalog number ab6586; Abcam Limited; RRID: AB_305584), rabbit polyclonal monospecific anti–collagen VII (1:10,000; same as for IHC), rabbit monoclonal anti–keratin 5 (1:1000; clone EP1601Y; catalog number ab52635; Abcam Limited; RRID: AB_869890), and mouse monoclonal anti–keratin 17 (1:1000; catalog number MA1-06325; Thermo Fisher Scientific; RRID: AB_559766) antibodies diluted in 2% bovine serum albumin in tris-buffered saline were incubated overnight at room temperature. The following day, tissue sections were incubated with the Alexa Fluor secondary antibodies from Thermo Fisher Scientific: goat anti-mouse IgG1 (A-21127; RRID: AB_2535769), F(ab')2-goat anti-rabbit IgG (H + L) (A-21246; RRID: AB_2535814), donkey anti-mouse IgG (H + L) (A-31570; RRID: AB_2536180), or goat anti-rabbit (A-32733; RRID: AB_2633282), all diluted 1:200 in phosphate-buffered saline for 1 hour together with DAPI at final concentration 1 ng/mL (catalog number MBD0015; Sigma Aldrich, St. Louis, MO) and mounted with DAKO fluorescent mounting medium (catalog number S3023; Agilent Technologies).

### Image Analysis of Tissue Sections

Whole-slide imaging was performed using the VS120 virtual microscopy slide scanning system (Olympus, Tokyo, Japan). Whole tissue sections were analyzed, and representative images were acquired using the software Qupath^34^ version 0.3.0 and ImageJ^35^ version 1.53j (NIH, Bethesda, MD; *https://imagej.net/ij*). After automatic cell detection, quantification of KRT5 and KRT17 was done using cell classifiers, and quantification of collagen VII was done using the pixel classifiers with set thresholds of positive staining. KRT5^+/–^ KRT17^+/–^ cell populations were calculated as a percentage of all detected cells from whole tissue sections. Collagen VII was quantified as a percentage of the area of collagen VII–positive pixels of the whole tissue area based on the DAPI channel with a set lower threshold to detect both cell nuclei and tissue autofluorescence to capture the most tissue. For quantification of frequencies of KRT17^+^ cells in close proximity to collagen VII–positive pixels, the distance of 20 μm was set as a compromise to detect as many cells from the pseudostratified epithelium as possible while avoiding detecting cells in the mucus filling the airways and honeycomb cysts. All tissue sections for the quantification were analyzed in the same way within the different combinations of markers.

### RNA *in Situ* Hybridization

Tissue sections (4 μm thick) from FFPE blocks derived from distal lung tissue of controls (*n* = 3, 1 FFPE block/individual) and patients with IPF (*n* = 3, 1 FFPE block/individual) were used for RNA *in situ* hybridization using RNAscope 2.5 HD Reagent Kit - RED (catalog number 322350; ACD Bio-Techne, Newark, CA), according to the manufacturer's instructions. Briefly, slides with tissue sections were incubated at 60°C for 60 minutes, followed by deparaffinization, rehydration, and incubation with hydrogen peroxide for 10 minutes. A combination of a heat-induced and an enzymatic antigen retrieval was performed by boiling in RNAscope Target Retrieval (ACD Bio-Techne) for 15 minutes and incubating with Protease Plus (ACD Bio-Techne) at 40°C for 30 minutes. Tissue sections were hybridized with the target probe for COL7A1 (catalog number 803381; ACD Bio-Techne) and a series of amplification solutions. Finally, the signal was detected with fast red dye, and tissue sections were counterstained with 50% Mayer hematoxylin (catalog number 01820; Histolab), and mounted with Pertex mounting medium (catalog number 00840-05; Histolab) and number 1 thickness cover glass. Whole-slide imaging was performed in the same manner as described above. After histologic analysis, representative images were acquired using the software Qupath version 0.3.0 and ImageJ version 1.53j.

### Culture of Human Lung Fibroblasts with TGF-β1 Stimulation

Primary lung fibroblasts were isolated from explanted distal lung tissue of controls (*n* = 6) and patients with IPF (*n* = 4), as previously described.^36^ Fibroblasts were expanded in Dulbecco's modified Eagle's medium (catalog number 31966047; Sigma Aldrich) supplemented with 0.5% gentamicin (catalog number G1272; Sigma Aldrich), 1% amphotericin B (catalog number A2942; Sigma Aldrich), 1% GlutaMAX (catalog number 35050061; Gibco, Thermo Fisher Scientific), and 10% Fetal Clone III Serum (catalog number SH30109.03; Cytiva, Marlborough, MA) at 37°C, 10% CO_2_ and were used for cell experiments in passage between 5 and 8. Fibroblasts were seeded on 96-well plates (250,000 cells/cm^2^) and allowed to attach to the bottom of well plates overnight. The following day before the transforming growth factor (TGF)-β1 stimulation, fibroblasts were starved in supplemented Dulbecco's modified Eagle's medium with 0.4% Fetal Clone III Serum at 37°C, 10% CO_2_ for 2 hours. Following starvation, cells were treated with TGF-β1 at final concentration of 10 ng/mL (catalog number 240-B; R&D Systems, Minneapolis, MN) or without in starvation medium for 48 hours at 37°C, 10% CO_2_. After treatment, cell lysates were harvested for RNA using RNeasy Mini Kit (catalog number 74104; Qiagen, Hilden, Germany) and measured on NanoDrop 2000c Spectrophotometer (Thermo Fisher Scientific), according to manufacturer's instructions.

### Quantitative RT-PCR Analysis

From extracted total RNA, cDNA was synthesized with QuantiTect Reverse Transcription kit (catalog number 205313; Qiagen), according to the manufacturer's instructions. Quantitative real-time PCR of cDNA samples was performed using primers, Quantifast SYBR PCR kit (catalog number 204056; Qiagen) and Mx3005P Real-Time PCR System (Agilent Technologies) or StepOne Plus Real-Time PCR System (instrument serial number 272001599; Applied Biosystems, Waltham, MA), according to manufacturers’ instructions. For COL7A1, forward primer 5′-GTGAGGACTGCCCCTGAG-3′ and reverse primer 3′-GACTCCACCTTCGAGACCC-5′ (Thermo Fisher Scientific), which amplify a 210-bp fragment spanning exons 17 to 19 [predicted to target *Homo sapiens* collagen type VII α 1 chain (*COL7A1*), transcript variant X1], were used. For other genes, the following primers from Qiagen (catalog number 249900) were used: Hs_COL1A1_1_SG [GeneGlobe identifier (ID) QT00037793], Hs_LAMB3_1_SG (GeneGlobe ID QT00084658), Hs_LAMC2_1_SG (GeneGlobe ID QT00085484), and Hs_ACTA2_1_SG (GeneGlobe ID QT00088102). The primer Hs_PPIA_1_SG (GeneGlobe ID QT00052311) was applied as the reference gene for calculating delta quantification cycle (ΔCq) values. ”No reverse transcription” and “no template” controls were included in the experiments. The 2^−ΔΔCq^ method was used for calculating the fold change of individual genes expressed in fibroblasts treated with TGF-β1 relative to the expression in untreated fibroblasts derived from the same individuals. Values of Cq > 35 were not included in the calculations as they were considered an unspecific amplified signal. Furthermore, three samples were excluded post-analysis because of concurrent high variance in PPIA Cq and high ACTA2 Cq (>30), probably resulting from low amount of input cDNA.

### Statistical Analysis

All graphs and statistical analyses of data were performed in GraphPad Prism version 9.3.1 (GraphPad Software, Boston, MA). The nonparametric *U*-test was used for bulk RNA-sequencing data and quantification of immunofluorescence stainings. For quantitative RT-PCR data, the one-sample *t*-test was applied. Values of *P* < 0.05 were considered as statistically significant. The visualized values and number of individuals/biological samples (*n*) for each group are reported in the figure description related to each analysis.

## Results

### Patient Characteristics

In this study, lung tissues from eight organ donors and eight patients with IPF were used, the clinical characteristics of which are summarized in Table 1. The group of control individuals consisted of seven males and one female, with a mean ± SD age of 60 ± 12 years. Four of them were never smokers, three were former smokers, and one was a current smoker. The group of patients with IPF included five males and three females, with a mean ± SD age of 60 ± 5 years, of which two were never smokers and six were former smokers. The use of individual samples in different experiments is depicted in Supplemental Table S1.

### Collagen VII Is Increased in IPF Lung Tissue

**Figure 2:**
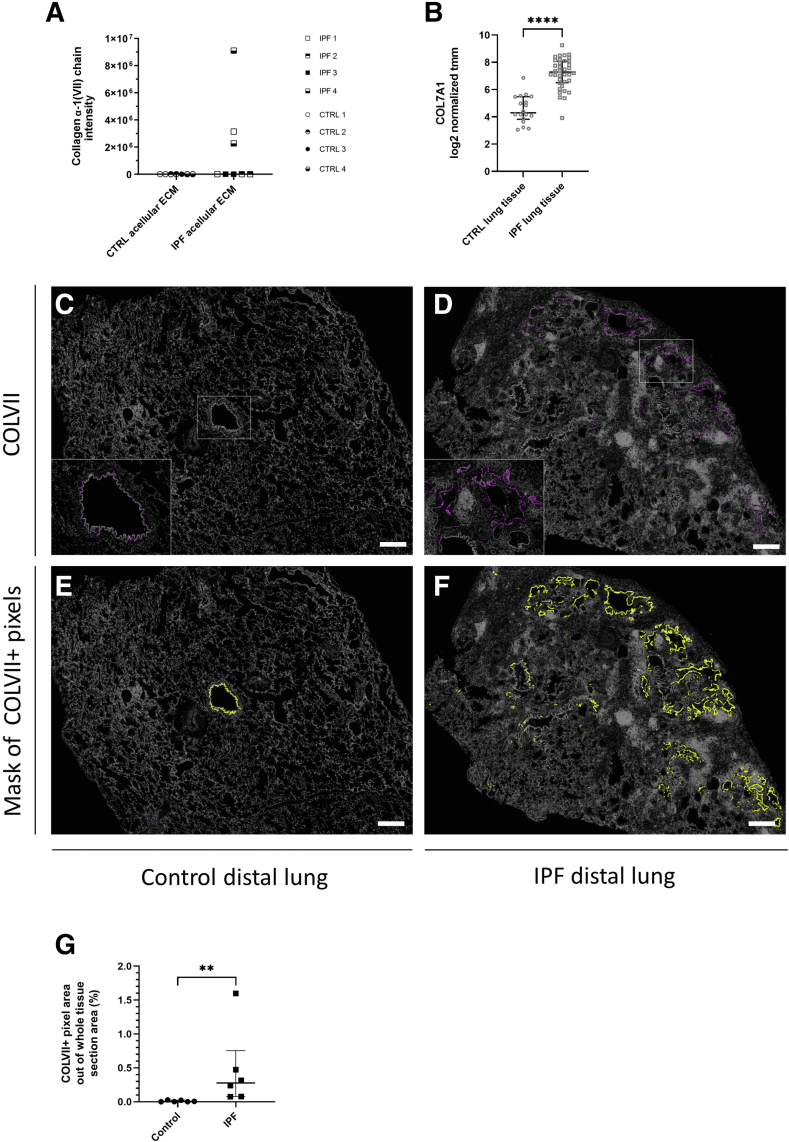
Collagen VII in distal lung tissue of controls (CTRL) and patients with idiopathic pulmonary fibrosis (IPF) detected by mass spectrometry, bulk RNA sequencing, and immunohistochemistry. **A:** Protein intensity of collagen α-1(VII) chain from mass spectrometry analysis in control acellular extracellular matrix (ECM; CTRL) and IPF acellular ECM (IPF) with two technical replicates/each individual. Data points on the *x* axis represent samples that were not detected in the analysis. **B:** COL7A1 mRNA expression in whole native lung tissue of CTRLs and patients with IPF derived from bulk RNA-sequencing dataset GSE134692^30^ (*https://www.ncbi.nlm.nih.gov/geo*) with the trimmed mean of M-values (tmm) normalization method used. Median and interquartile range are shown for each group. Statistical analysis: *U*-test. **C**–**F:** Representative images of control (**C** and **E**) and IPF (**D** and **F**) distal lung tissue sections with stained collagen VII (COLVII; magenta color in **C** and **D**, with magnified areas with an airway in **C** and airways and honeycombing in **D** depicted in the **left** bottom corner) and a corresponding mask of detected positive collagen VII pixels (yellow color in **E** and **F**). **G:** Quantification of positive collagen VII (COLVII^+^) pixel area in whole tissue sections of CTRL and IPF distal lung tissue. Median and interquartile range are shown for each group. Statistical analysis: *U*-test. *n* = 4 CTRL and IPF (**A**); *n* = 18 CTRL (**B**); *n* = 36 IPF (**B**); *n* = 6 CTRL and IPF (**G**). ∗∗*P* < 0.01, ∗∗∗∗*P* ≤ 0.0001. Scale bar = 1 mm (**C**–**F**).

Collagen α-1(VII) chain was identified in a previously published, re-examined proteomic data set^4^ in acellular IPF lung ECM (detected in two of four patients), whereas no signal of collagen α-1(VII) chain was detected in acellular ECM derived from controls (Figure 2A). In support of this observation, analysis of collagen VII gene expression in the public RNA-sequencing data set (GSE134692)^30^ revealed a significantly higher expression of COL7A1 in IPF lung tissue compared with control tissue (*P* < 0.0001) (Figure 2B).

**Supplemental Figure S3:**
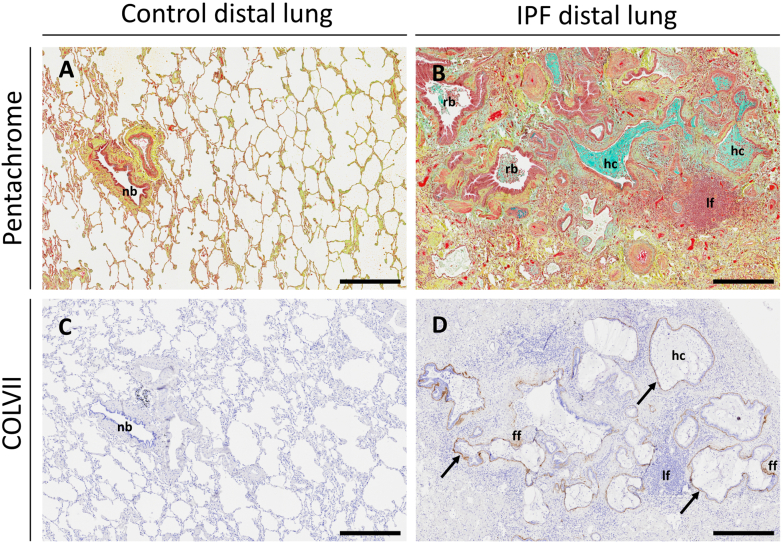
Histomorphology of control and idiopathic pulmonary fibrosis (IPF) distal lungs. Images of modified Russel-Movat pentachrome (**A** and **B**) and immunohistochemistry staining of collagen VII protein (COLVII; **arrows**; **C** and **D**) of similar regions from control [**A** and **C**; normal bronchiole (nb)] and IPF (**B** and **D**) distal lungs enriched with remodeled bronchioles (rb), subpleural honeycombing (hc), fibroblast foci (ff), and lymphoid follicles (lf). Pentachrome highlights elements of connective tissue (collagen, yellow; elastic fibers, black; and mucins, green to blue). Scale bar = 500 μm (**A**–**D**).

To explore collagen VII *in situ*, lung tissue sections from controls and patients with IPF were examined using H&E staining, pentachrome staining, and immunohistochemistry (Figure 2, C–F, and Supplemental Figure S3). The control distal lung tissue sections exhibited normal parenchyma with thin alveolar walls and a limited number of airways (Figure 2C and Supplemental Figure S3A). Distal parenchymal regions of IPF lungs close to the visceral pleura displayed the histopathologic pattern typical for UIP—the lung parenchymal structure was completely altered, with most of the alveoli being replaced by cell- and ECM-rich tissue, fibroblast foci, and honeycombing (Supplemental Figure S3B). Furthermore, smooth muscle cell hyperplasia, immune cell infiltration, and hyperplasia were commonly observed in IPF. The IPF tissue also exhibited a striking increase of airway and airway-like epithelial structures, such as normal-looking bronchioles, enlarged or remodeled bronchioles, and honeycomb cysts with or without mucous content. Basement membrane–like staining of collagen VII was found surrounding these epithelial structures (Figure 2, D and F, and Supplemental Figure S3D). In the control sections, collagen VII appeared only in bronchioles with various amounts depending on the size of the airway (Figure 2, C and E, and Supplemental Figure S3C). Quantification of collagen VII–positive pixels (mask of detected positive pixels shown in Figure 2, E and F) demonstrated a significant increase of collagen VII protein in the IPF lung tissue sections in comparison to the tissue sections from controls (*P* = 0.0043) (Figure 2G). Altogether, the data suggested that collagen VII constitutes a structural component of the lung ECM, which is up-regulated in IPF.

### Collagen VII Is Abundant in Pathologic Epithelial and Fibroblast Structures in IPF Lungs

**Figure 3:**
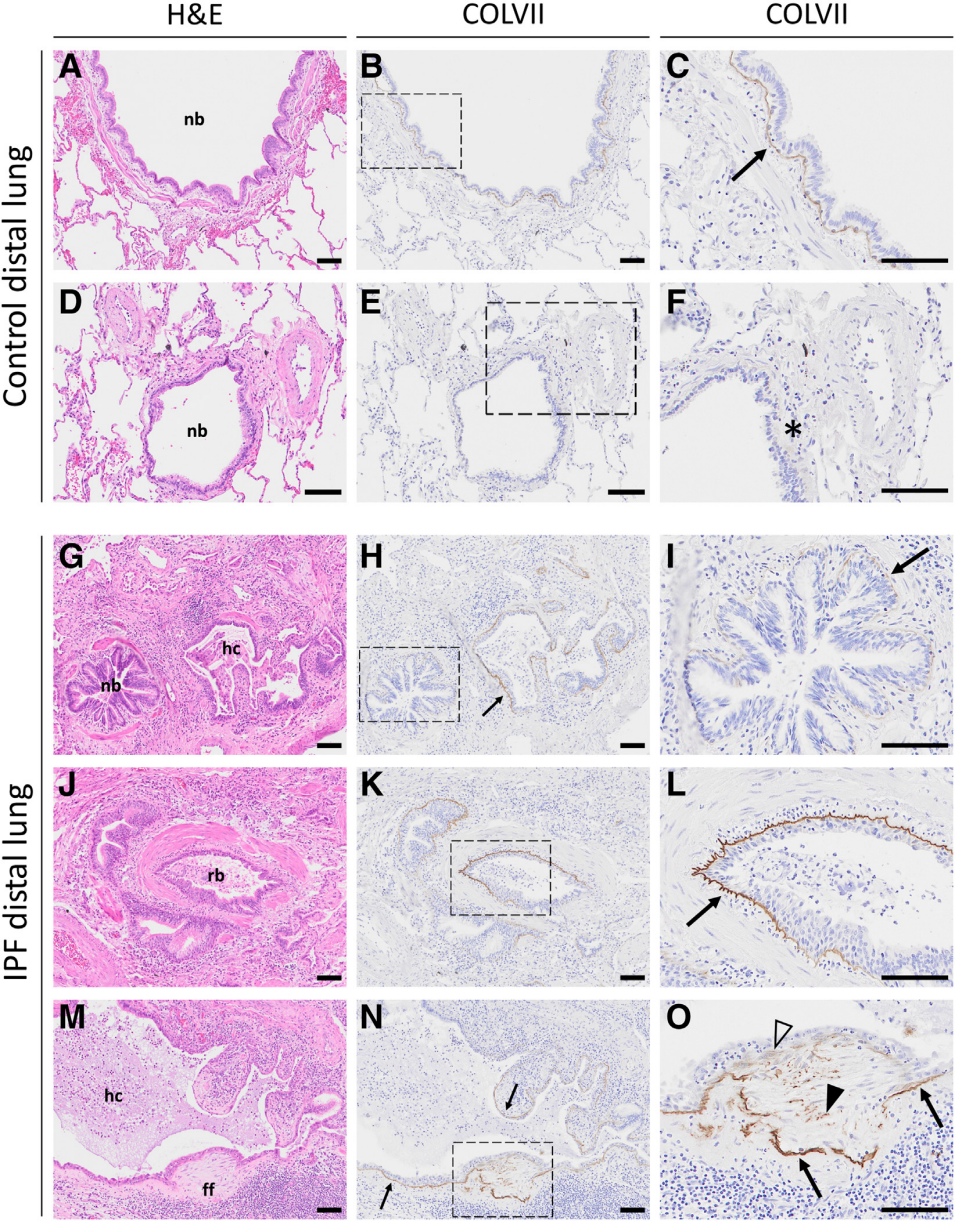
Spatial protein distribution of collagen VII (COLVII) in distal lungs. Images of hematoxylin and eosin (H&E) staining (**A**, **D**, **G**, **J**, and **M**) and immunohistochemistry staining of collagen VII protein (brown; **B**, **C**, **E**, **F**, **H**, **I**, **K**, **L**, **N**, and **O**) performed on consecutive sections of distal lung tissue of patients with idiopathic pulmonary fibrosis (IPF; **G**–**O**) and controls (**A**–**F**), with magnified images of collagen VII expression in the basement membrane zone of larger bronchiole (**C**) or absent protein in small bronchiole in control lung (**F**). Tissue sections of IPF lungs show normal-looking bronchiole (nb; **G**–**I**), remodeled bronchiole with infiltrated neutrophils in the basement membrane zone (rb; **J**–**L**), honeycomb cysts (hc; **G**, **H**, **M**, and **N**), and fibroblast focus (ff; **M**–**O**). **Arrows** indicate collagen VII in the basement membrane zone, **solidarrowheads** indicate extracellular collagen VII, **empty arrowheads** indicate cells with intracellular collagen VII, and **asterisk** indicates absent staining. Scale bar = 100 μm (**A**–**O**).

**Supplemental Figure S4:**
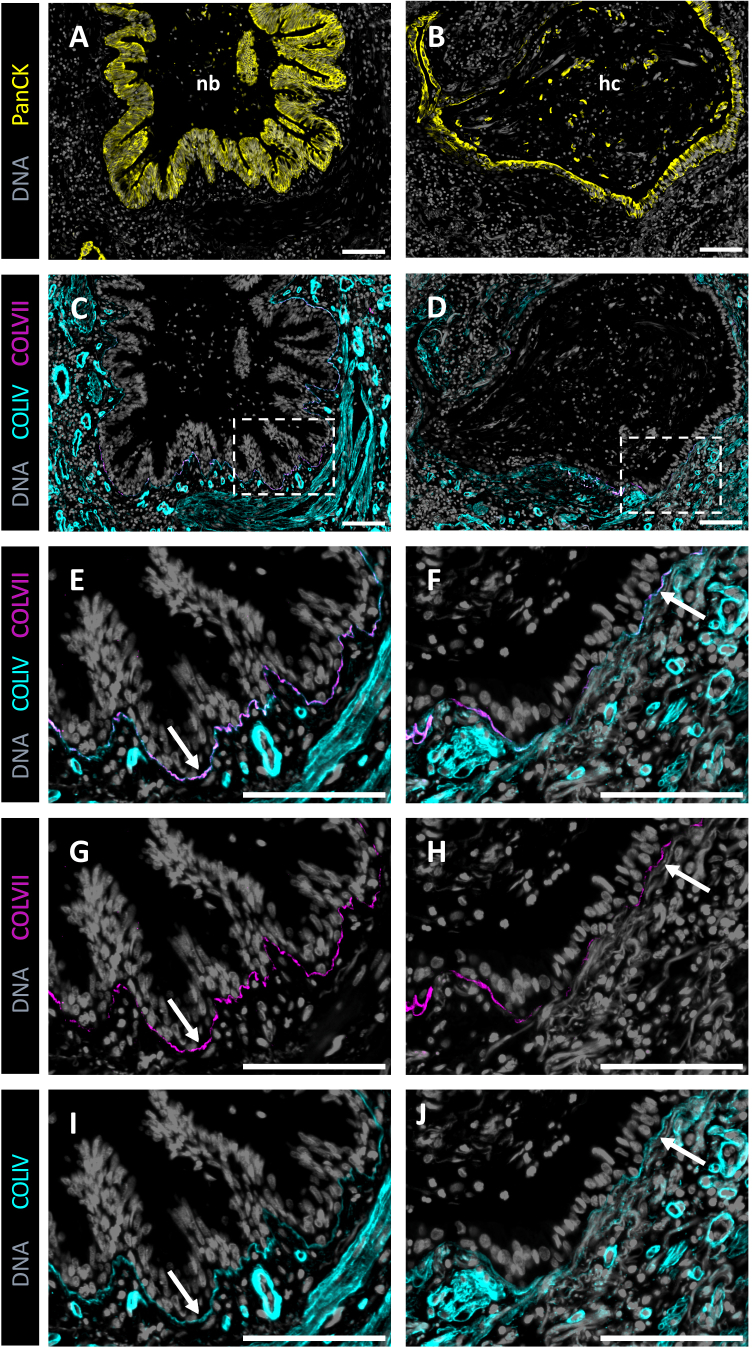
Colocalization of collagen VII (COLVII) and collagen IV (COLIV) in the basement membrane zone. Images of immunofluorescence staining of pan-cytokeratin (PanCK) visualizing epithelial cells and co-staining of COLIV and COLVII proteins in the basement membrane zone (**arrows**) of normal-looking bronchiole (nb; **A**, **C**, **E**, **G**, and **I**) and honeycomb cyst (hc; **B**, **D**, **F**, **H**, and **J**) in idiopathic pulmonary fibrosis distal lung. Nuclei are stained with DAPI (DNA). Scale bar = 100 μm (**A**–**J**).

To investigate the spatial distribution of collagen VII protein more closely, first, parenchymal regions were examined with IHC. In control distal lung tissue sections (Figure 3, A–F), collagen VII was found only in the basement membrane zone of bronchioles (Figure 3, B and C). The bronchioles exhibited a heterogeneous pattern of collagen VII expression; in most cases, they showed weak or absent staining (Figure 3, E and F). In general, the staining intensity appeared to reduce with decreasing airway size and the associated transition from bronchiolar pseudostratified epithelium to simple columnar and cuboidal epithelium. In normal-looking bronchioles in distal regions of IPF lungs, collagen VII was detected to a similar extent as in the control lungs (Figure 3, H and I). However, the most prominent collagen VII extracellular deposition was seen in the basement membrane zone of remodeled bronchioles (Figure 3, K and L) and under hyperplastic bronchiolar-like columnar to squamous epithelium in honeycomb cysts (Figure 3, H and N). In the basement membrane, collagen VII colocalized with collagen IV (Supplemental Figure S4). Interestingly, collagen VII was also found in many, but not all, fibroblastic foci (Figure 3, N and O). The adjacent tissue sections for Figure 3, B–C, E–F, H–I, K–L, and N–O, stained with H&E, are shown in Figure 3, A, D, G, J, and M. Collagen VII staining was not present in blood vessels, dense fibrotic areas, areas with retained alveolar structures with thickened alveolar walls, or normal-looking alveolar regions.

**Supplemental Figure S5:**
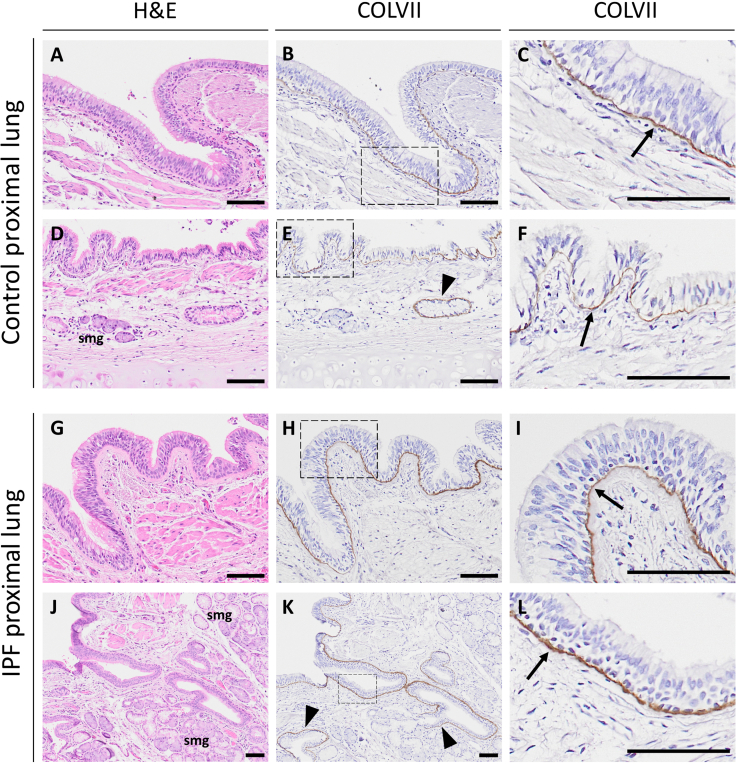
Spatial protein distribution of collagen VII (COLVII) in proximal lungs. Images of hematoxylin and eosin (H&E) staining (**A**, **D**, **G**, and **J**) and immunohistochemistry staining of collagen VII protein (**B**, **C**, **E**, **F**, **H**, **I**, **K**, and **L**) performed on consecutive sections of proximal lung tissue of controls (**A**–**F**) and patients with idiopathic pulmonary fibrosis (IPF; **G**–**L**), with magnified images of the bronchiolar pseudostratified epithelium lined by collagen VII (**C**, **F**, **I**, and **L**). **Arrows** indicate collagen VII in the basement membrane zone of airway, and **solidarrowheads** indicate collagen VII in the basement membrane zone of the duct of submucosal glands. Scale bar = 100 μm (**A**–**L**). Smg, submucosal glands.

In addition, tissue sections from proximal regions of the lung were examined to investigate any difference in collagen VII deposition in bronchi between controls and patients with IPF. In control lung tissue (Supplemental Figure S5, A–F), collagen VII protein was detected in the upper part of the basement membrane located under the bronchial pseudostratified epithelium (Supplemental Figure S5, B, C, E, and F). In addition, collagen VII was seen lining ducts of submucosal glands with continuing pseudostratified epithelium, with a decreasing collagen VII intensity toward the secretory part of the glands (Supplemental Figure S5E). The proximal regions of IPF lungs (Supplemental Figure S5, G–L) did not differ from controls in terms of the presence of collagen VII. The protein was also found in the basement membrane zone of bronchi (Supplemental Figure S5, H and I) and glandular ducts (Supplemental Figure S5K). As such, collagen VII changes in IPF lungs appeared restricted to aberrant epithelial structures and fibroblast foci.

### IPF Distal Lungs Display an Increase in Basal and Abnormal Basal Cell Populations Expressing COL7A1

**Figure 4:**
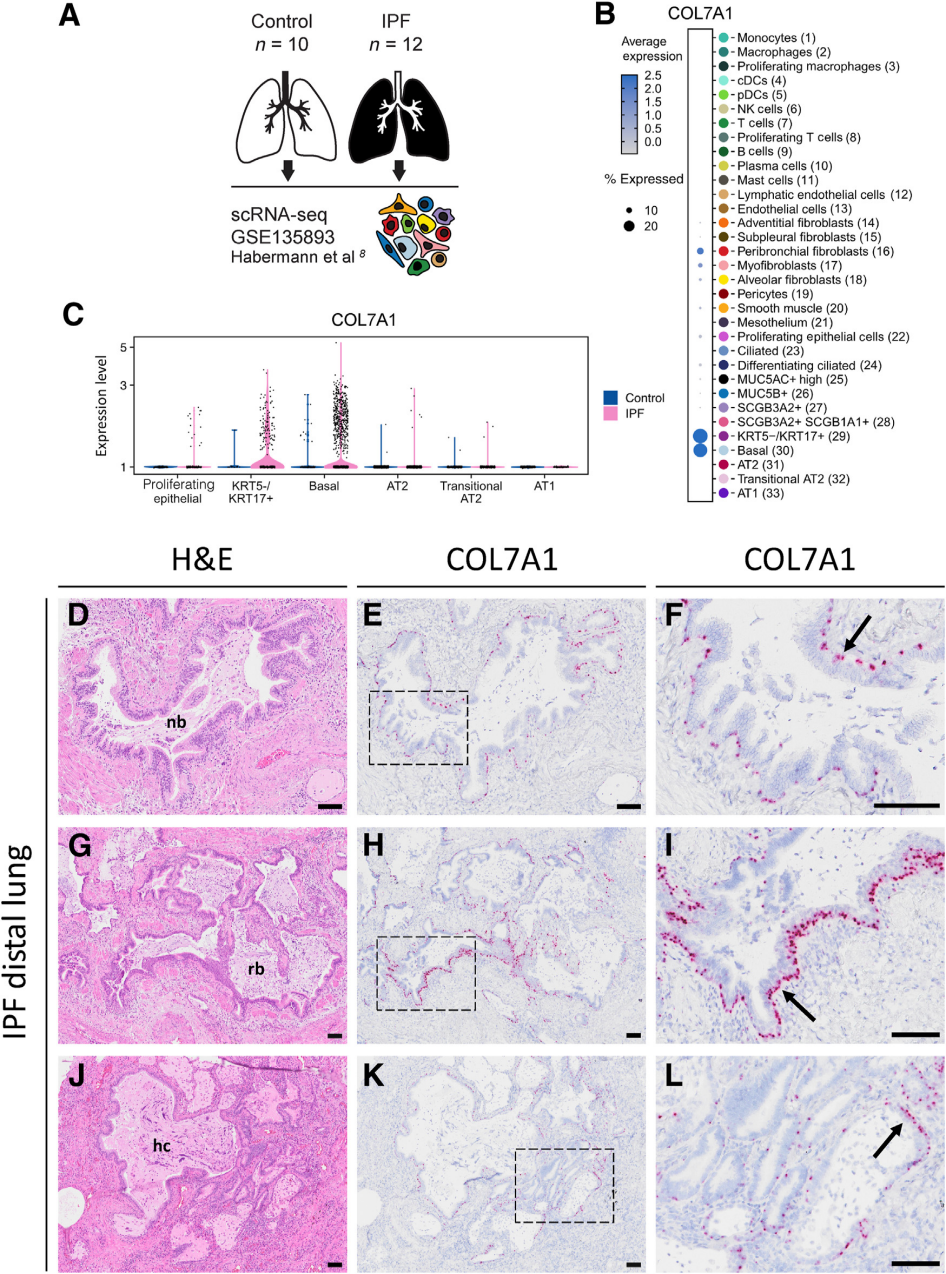
Transcriptomic analysis and spatial distribution of COL7A1 mRNA in distal lungs. **A:** Re-analysis of public single-cell RNA-sequencing (scRNA-seq) data set from Habermann et al^8^ (*https://www.ncbi.nlm.nih.gov/geo*; accession number GSE135893). **B:** Normalized COL7A1 mRNA expression level among all identified cell types in both control and idiopathic pulmonary fibrosis (IPF) lungs. **C:** Violin plot of log normalized COL7A1 mRNA expression level within selected epithelial cell populations in control (blue) and IPF lungs (pink). **D**–**L:** Images of hematoxylin and eosin (H&E) staining (**D**, **G**, and **J**) and *in situ* hybridization (**E**, **F**, **H**, **I**, **K**, and **L**) of COL7A1 mRNA on consecutive sections of distal lung tissue of patients with IPF, showing COL7A1^+^ epithelial cells localized to normal-looking bronchiole (nb; **E** and **F**), remodeled bronchiole (rb; **H** and **I**), and honeycomb cysts (hc; **K** and **L**). **Arrows** indicate COL7A1^+^ epithelial cells. Scale bar = 100 μm (**D**–**L**). AT1, alveolar type I cell; AT2, alveolar type II cell; cDCs, classical dendritic cells; KRT, keratin; MUC5AC, mucin 5AC; MUC5B, mucin 5B; NK, natural killer; pDCs, plasmacytoid dendritic cells; SCGB1A1, secretoglobin family 1A member 1; SCGB3A2, secretoglobin family 3A member 2.

To pinpoint a cellular source of collagen VII, a public single-cell RNA-sequencing data set with cells from 10 control and 12 IPF lungs (GSE135893), generated by Habermann et al,^8^ was partially re-annotated and analyzed (Figure 4, A–C). Of all cell types in the control and IPF lungs, epithelial basal cells and aberrant basaloid cells, which are negative for KRT5 but positive for KRT17 (KRT5^–^/KRT17^+^), showed the most prominent expression of COL7A1 mRNA (Figure 4B). Furthermore, the expression of COL7A1 mRNA was increased in these basal cell populations in IPF compared with the control lungs (Figure 4C). Expression of mRNAs for proteins involved in anchoring (LAMA3, LAMB3, and LAMC2) and hemidesmosomal complexes (ITGA6, ITGB4, PLEC, CD151, DST, and COL17) was up-regulated in the basal cell populations from IPF compared with that in controls (Supplemental Figure S6). Interestingly, mRNA expression of the transmembrane collagen XVII (COL17A1) was also increased and specific for basal cell populations in IPF, similar to that in COL7A1.

To evaluate the cellular mRNA expression of COL7A1 *in situ*, RNAscope *in situ* hybridization was performed in distal lung tissue sections (Figure 4, E, F, H, I, K, L, with adjacent sections stained with H&E in D, G, and J; and Supplemental Figure S7, A, B, D, E, G, H, J, and K, with adjacent sections stained with H&E in C, F, and I). IPF distal lungs showed an overall increased area enriched with epithelial structures with COL7A1^+^ cells compared with that in controls (Supplemental Figure S7, A and B). In the epithelium of normal-looking bronchi and bronchioles present in the distal lung tissue sections, the pattern of COL7A1 mRNA distribution was similar to that in controls (Figure 4, E and F, and Supplemental Figure S7, D, E, G, H, J, and K). However, COL7A1^+^ cells appeared more abundant in the aberrant hyperplastic epithelium of remodeled bronchioles (Figure 4, H and I). Furthermore, COL7A1^+^ cells were also observed at the base of honeycomb cysts (Figure 4, K and L), in cells in fibroblast foci, and, to a lesser extent, in a few scattered vascular smooth muscle cells, airway smooth muscle cells, and cells in the dense fibrotic tissue (data not shown).

Control distal lung tissue sections contained a few bronchi, which displayed many COL7A1^+^ epithelial cells at the base of the pseudostratified epithelium (Supplemental Figure S7, D and E), whereas bronchioles in distal lung tissue sections showed a reduction in COL7A1^+^ epithelial cells with decreasing bronchiole size (Supplemental Figure S7, G, H, J, and K). Furthermore, scattered vascular smooth muscle cells and airway smooth muscle cells expressed COL7A1, but to a lesser extent compared with epithelial cells, whereas alveoli appeared mostly negative.

**Figure 5:**
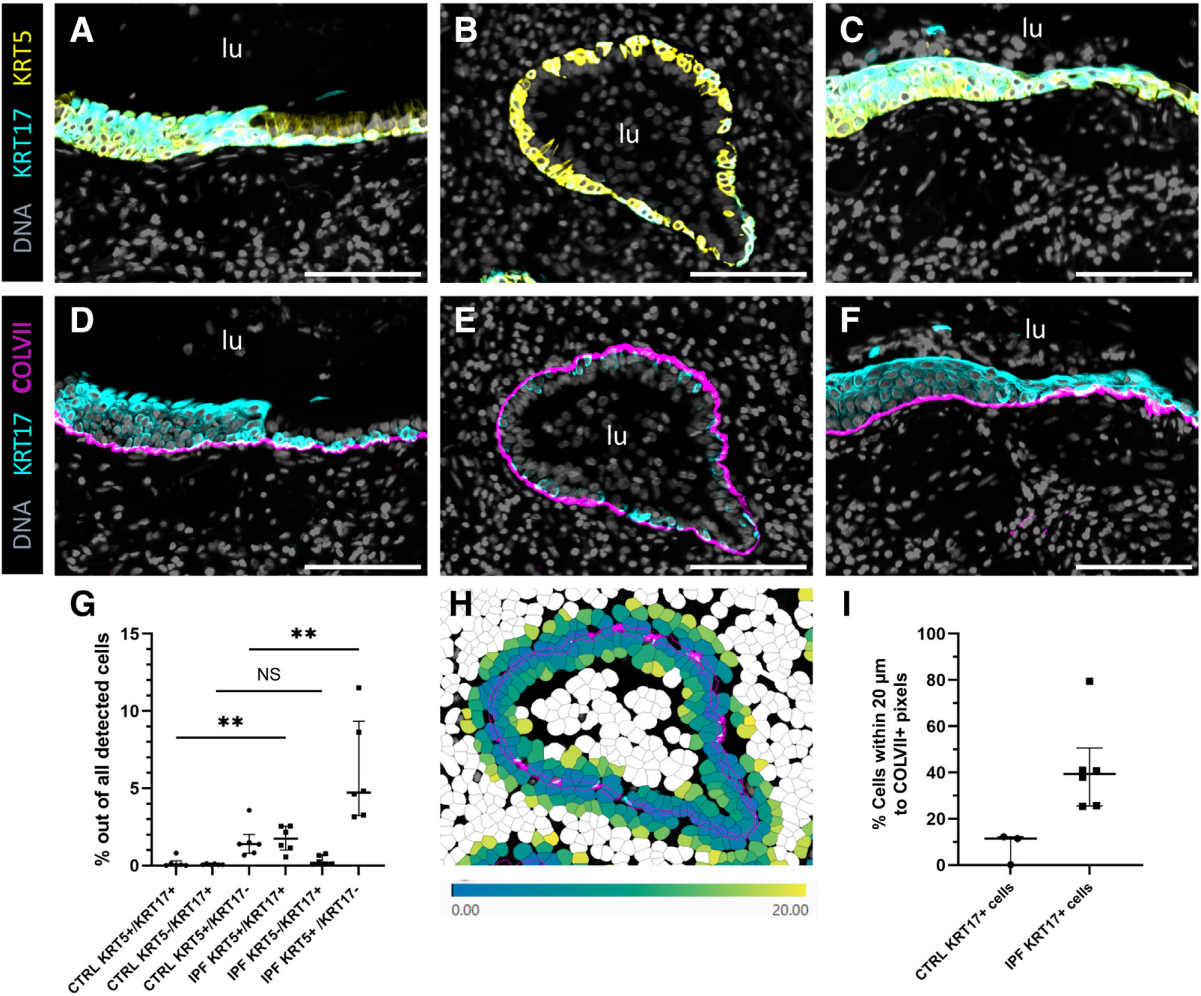
Spatial localization of keratin 5 (KRT5), keratin 17 (KRT17), and collagen VII (COLVII) in idiopathic pulmonary fibrosis (IPF) distal lungs. **A**–**F:** Images of consecutive sections of IPF distal lung, showing immunofluorescence costaining of proteins KRT17 (**A**–**C**) with KRT5 (**A**–**C**), and KRT17 (**D**–**F**) with COLVII (**D**–**F**) in remodeled bronchioles, highlighting the changes in the morphology of cells. Nuclei are stained with DAPI (DNA). **G:** Quantification of different basal cell populations in whole tissue sections [IPF and control (CTRL)]. Median and interquartile range are shown for each group. Statistical analysis: *U*-test. **H:** Schematic picture of cells within 20 μm from detected COLVII^+^ pixels (magenta). **I:** Percentage of KRT17^+^ cells within 20 μm from COLVII^+^ pixels in CTRL and IPF distal lung tissue sections. Percentage data points of from three controls are not shown as no KRT17^+^ cells or less than three KRT17^+^ cells were detected in the lung tissue sections. Median and interquartile range are shown for each group. *n* = 6 IPF and CTRL (**G** and **I**). ∗∗*P* < 0.01. Scale bars = 100 μm (**A**–**F**). lu, lumen; NS, nonsignificant.

**Supplemental Figure S8:**
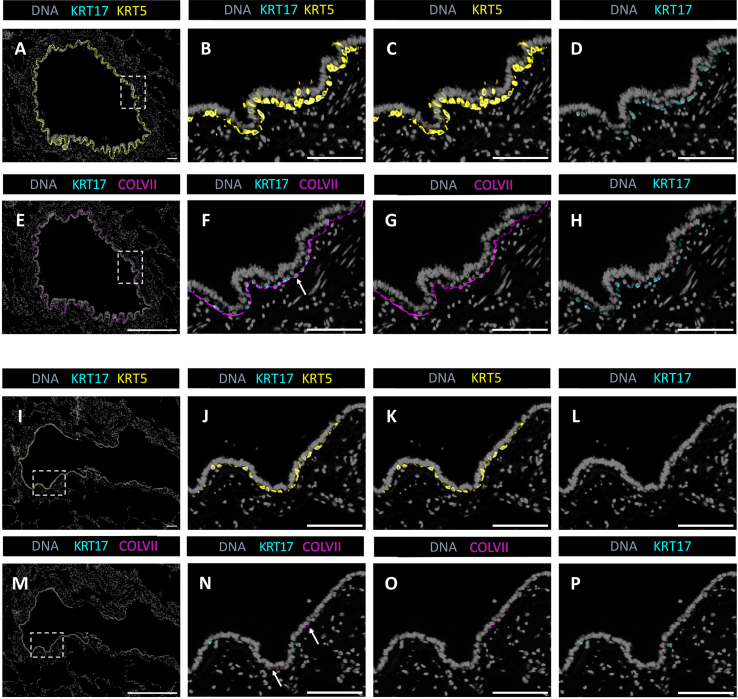
Spatial localization of collagen VII (COLVII), keratin 5 (KRT5), and keratin 17 (KRT17) in control distal lung. Images of consecutive sections of control distal lung tissue, showing immunofluorescence costaining of KRT17 with KRT5, and KRT17 with COLVII (**arrows**) in larger bronchiole (**A**–**H**) and smaller bronchiole (**I**–**P**). Nuclei are stained with DAPI (DNA). Scale bar = 100 μm (**A**–**P**).

Immunofluorescence staining of KRT5 and KRT17 proteins was performed for spatial localization of the basal cell populations. It demonstrated a large abundance of different basal cell populations, highlighting a pronounced re-epithelialization of the fibrotic lung (Figure 5). In bronchioles surrounded by collagen VII (Supplemental Figure S8, E–G, and M–O) in control lungs and some parts of epithelium in remodeled bronchioles in IPF lungs, both keratins seemed to label cells at the base of the pseudostratified epithelium, and KRT5 was additionally expressed in a few scattered columnar cells, similar to that in controls [Figure 5, A, B, D, and E (for IPF lungs), and Supplemental Figure S8, A–F, H–N, and P (for control lungs)]. In contrast, hyperplastic squamous epithelium of remodeled airways and honeycomb cysts exhibited an elevated expression of both keratins (Figure 5, A, C, D, and F). Quantification of different basal cell populations (Figure 5G) showed a significant increase of KRT5^+^/KRT17^+^ (*P* = 0.0043) and KRT5^+^/KRT17^–^ (*P* = 0.0087) populations in IPF distal lung tissue sections compared with control lung tissue sections, whereas there was no statistically significant difference in KRT5^–^/KRT17^+^ population between the groups (*P* = 0.0628). Further quantification of frequencies of KRT17^+^ cells of all cells within 20 μm from collagen VII–positive pixels (schematic picture of cells within 20 μm in Figure 5H) showed that the percentage of KRT17^+^ cells was increased in IPF lungs (median, 39.4%; Figure 5I) compared with that in control lungs (median, 11.5%; Figure 5I), indicating more abundant KRT17^+^ cells in close proximity to collagen VII. Altogether, COL7A1 appeared to be expressed primarily by epithelial cell clusters, such as basal cells and KRT5^–^/KRT17^+^ cells, in both control and IPF lung sections, where the overall expression was increased in IPF, due to a combination of increased number of basal cell populations and their increased cellular expression.

### COL7A1 Is Also Up-Regulated in Mesenchymal Cells and Regulated by TGF-β1 *in Vitro*

**Figure 6:**
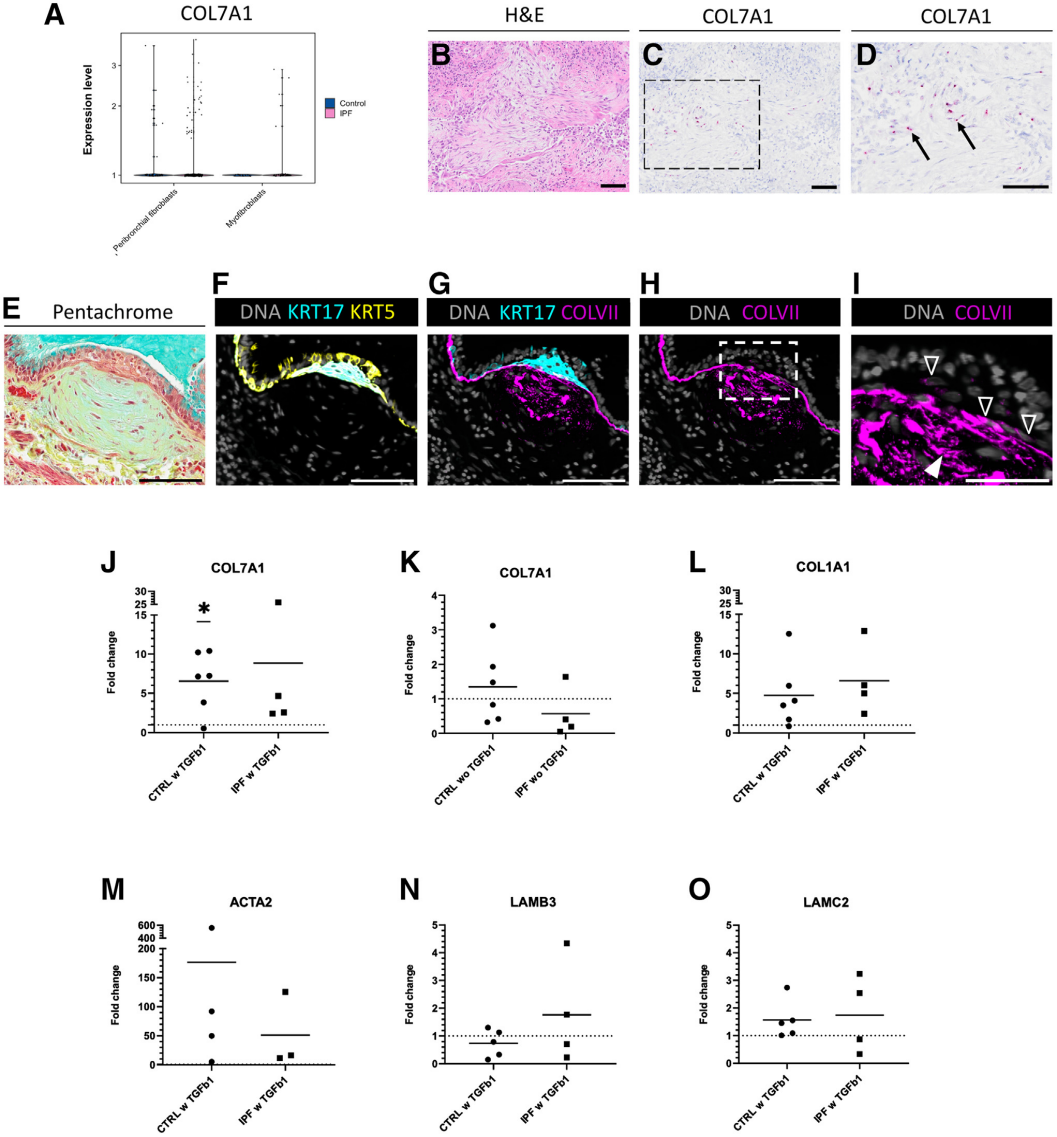
COL7A1 mRNA expression in mesenchymal cells. **A:** Normalized COL7A1 mRNA expression level within peribronchial fibroblasts and myofibroblasts clusters in control (CTRL; blue; **left**) and idiopathic pulmonary fibrosis (IPF) lungs (pink; **right**). **B**–**D:** Images of consecutive sections of IPF distal lung showing fibroblast focus stained with hematoxylin and eosin (H&E; **B**) and COL7A1 mRNA detected by *in situ* hybridization (**C**, with detail in **D**). **E**–**I:** Images of another fibroblast focus stained with pentachrome (**E**) and collagen VII (COLVII; **G** and **H**, with detail in **I**) in relation to keratin 5 (KRT5; **F**) and keratin 17 (KRT17; **F** and **G**). Nuclei are stained with DAPI (DNA). **Arrows** indicate COL7A1 mRNA, **empty arrowheads** indicate cells with intracellular collagen VII, and **solidarrowheads** indicate extracellular collagen VII. **J**–**O:** Relative expression of mRNA COL7A1 (**J** and **K**), COL1A1 (**L**), ACTA2 (**M**), LAMB3 (**N**), and LAMC2 (**O**) in primary human lung fibroblasts derived from CTRLs and patients with IPF. Data are presented as fold change (FC; 2^−ΔΔCq^) of mRNA expression at 48 hours after transforming growth factor (TGF)-β1 treatment (w TGFb1) normalized to untreated fibroblasts within the same individuals (represented by **dashed line** at *y* = 1), except for **K**, where COL7A1 is presented as FC (2^−ΔΔCq^) of mRNA expression in untreated control (CTRL wo TGFb1) or IPF (IPF wo TGFb1) fibroblasts normalized to the mean mRNA expression in untreated control fibroblasts (represented by **dashed line** at *y* = 1). Mean is shown for each group. Statistical analysis: parametric one-sample *t*-test. *n* = 6 CTRL (**J**–**O**); *n* = 4 IPF (**J**–**O**). ∗*P* ≤ 0.05. Scale bars = 100 μm (**B**–**I**).

**Supplemental Figure S9:**
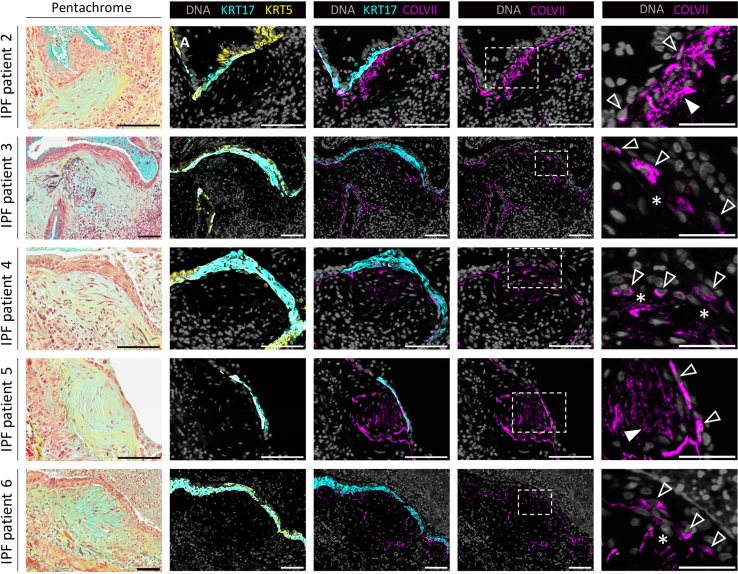
Expression of collagen VII (COLVII) and keratins in regions with fibroblast foci. Images of consecutive sections of idiopathic pulmonary fibrosis (IPF) distal lung tissue from all patients with IPF showing pentachrome (column 1), and immunofluorescence costaining of keratin 17 (KRT17) with keratin 5 (KRT5), and KRT17 with COLVII in fibroblast foci. Nuclei are stained with DAPI (DNA). Column 5 shows a detail of cells intracellularly positive for COLVII (**empty arrowheads**), missing or impaired extracellular COLVII in the basement membrane zone underneath (**asterisk**), and extracellular COLVII within fibroblast foci (**solidarrowheads**). Scale bars: 100 μm (**columns 1–4**); 50 μm (**column 5**).

The re-analysis of the public single-cell RNA-sequencing data set furthermore showed that COL7A1 mRNA was expressed in mesenchymal subsets, such as peribronchial fibroblasts and myofibroblasts (Figure 6A). This finding was consistent in fibroblast foci in the IPF distal lung, indicating the presence of COL7A1 mRNA (Figure 6, C and D) and collagen VII protein (Figure 6, F–I). The adjacent sections for Figure 6, C and D, were stained with H&E (Figure 6B), and the adjacent sections for Figure 6, F–I, were stained with pentachrome (Figure 6E). Interestingly, at the interface of many fibroblast foci and abnormal squamous epithelium, the inner layer of epithelial cells was intracellularly positive for collagen VII protein, often seen with disorganized or missing basement membrane underneath, a feature observed only at this location (Figure 6, G–I, and Supplemental Figure S9).

TGF-β1 is highly active in IPF and promotes fibroblast activation and ECM production; therefore, fibroblasts derived from lung tissue of controls (control fibroblasts) or patients with IPF (IPF fibroblasts) were subjected to 10 ng/mL TGF-β1 for 48 hours to investigate whether it up-regulates mRNA expression of COL7A1 and other relevant genes. COL7A1 was up-regulated in response to TGF-β1 with mean fold change (FC) = 6.6 (*P* = 0.02) for control fibroblasts (Figure 6J) compared with corresponding untreated fibroblasts (FC = 1). For IPF fibroblasts, no statistically significant difference was observed despite a trend in the gene up-regulation with mean FC = 8.9 (Figure 6J). When looking at the COL7A1 baseline mRNA expression of untreated cultured cells, IPF fibroblasts displayed a trend toward lower baseline mRNA expression with mean FC = 0.6 (Figure 6K) compared with the baseline mRNA expression of untreated control fibroblasts with mean FC = 1.4. mRNA expression of collagen type I α 1 chain (COL1A1) showed a trend in being up-regulated following TGF-β1 treatment with mean FC = 4.8 (*P* = 0.08) for control fibroblasts (Figure 6L) and mean FC = 6.6 (*P* = 0.09) for IPF fibroblasts (Figure 6L) compared with corresponding untreated fibroblasts (FC = 1). α-Smooth muscle actin (encoded by gene *ACTA2*), a component of the cytoskeleton, is a marker of fibroblast activation to a contractile, ECM-producing phenotype. Both control and IPF fibroblasts exhibited a prominent trend toward ACTA2 up-regulation; however, this was not statistically significant (FC > 5 in all stimulated cells) (Figure 6M), with mean FC = 176.6 for control fibroblasts and mean FC = 51.2 for IPF fibroblasts compared with unstimulated cells (FC = 1). Furthermore, no clear differences were seen in LAMB3 and LAMC2 mRNA (genes for chains of laminin 332, the protein-binding collagen VII) following TGF-β1 treatment in either control nor IPF fibroblasts (Figure 6, N and O). These data imply that collagen VII is also produced by mesenchymal cell populations and that its expression can be triggered in a profibrotic environment.

## Discussion

**Figure 7:**
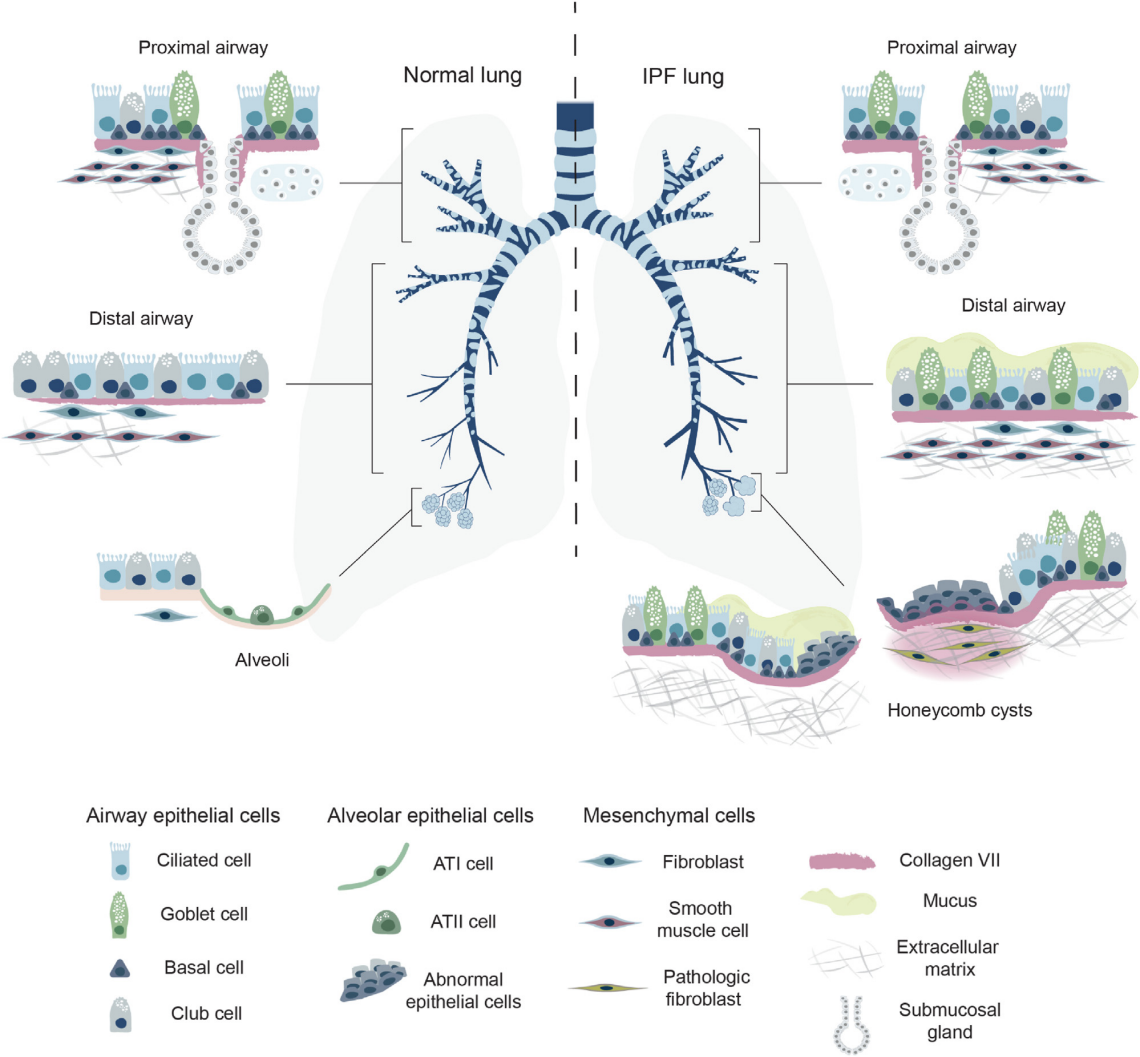
Summary of results in this study. While collagen VII is expressed to a similar extent in the basement membrane zone of bronchi in the control and the idiopathic pulmonary fibrosis (IPF) proximal lungs, the IPF distal fibrotic lungs display abundant pathologic structures, such as enlarged and remodeled bronchioles and honeycomb cysts, which are surrounded by collagen VII in their basement membrane zone. In contrast, in the control distal lungs, collagen VII localizes only to larger bronchioles. Furthermore, collagen VII was found in many IPF fibroblast foci and intracellularly in epithelial cells overlying these structures. Illustration made by Lisa Karlsson (Lung Biology Group, Lund University, Lund, Sweden). ATI, alveolar type I cell; ATII, alveolar type II cell.

Despite the reported increase in total lung collagen content in pulmonary fibrosis, little is known about the turnover and spatial localization of less abundant collagen types in normal lung tissue and different IPF niches. In this study, elevated levels of collagen VII were discovered in native IPF distal lung tissue compared with the control lung tissue. Further investigations revealed that in IPF, collagen VII protein was spatially restricted to the basement membrane zone of abundant pathologic epithelial structures, such as honeycomb cysts and remodeled airways, in addition to fibroblast foci, while not present in the vascular basement membrane zone. Basal cell populations were identified as the main cell types expressing COL7A1 mRNA, and their numbers were also increased in IPF distal lungs, colocalizing with collagen VII protein (summarized in Figure 7). Furthermore, COL7A1 mRNA was expressed by some subsets of mesenchymal cells and was up-regulated in primary fibroblasts by TGF-β1 fibrotic stimuli.

The analysis of transcriptomic data sets in larger cohorts of patients with IPF and controls validated the initial finding of elevated collagen VII levels, at the mRNA level.^8^^,^^30^ Consistent with these results, increased collagen VII was observed in a mouse model of IPF, where mice received an intratracheal injection with bleomycin compared with phosphate-buffered saline–treated controls.^37^ In another study, the collagen α-1(VII) chain was significantly up-regulated in the insoluble fraction enriched with ECM proteins in an ILD group, compared with controls.^38^ Furthermore, elevated levels of a specific collagen VII fragment have been detected in the serum of patients with systemic sclerosis and chronic obstructive pulmonary disease compared with controls.^39^ Interestingly, this suggests that collagen VII turnover can be measured systemically in diseases affecting the lung.

The exact cause of IPF is unknown; nevertheless, IPF is believed to initiate with repeated microinjuries to aging alveolar type II (ATII) cells, whose altered transcription program activates other cell types, including fibroblasts, to produce excessive amounts of ECM, including collagens. Several collagen types have been reported to be up-regulated in the fibroblast foci, such as collagen I, III, IV, V, VI, and XII, in addition to other ECM components (eg, fibronectin, hyaluronan, and versican).^15^^,^^40^ Furthermore, collagen VII protein was observed in many fibroblast foci of the IPF lungs (Figure 6, G–I, and Supplemental Figure S9). In support of this localization, the collagen α-1(VII) chain has been identified in a proteomic analysis of laser-capture microdissected fibroblast foci, as well as in some samples isolated from fibrotic and nonfibrotic alveoli.^15^ Using the same technique, Kamp et al^41^ showed COL7A1 among the most significantly up-regulated mRNA in fibroblast foci in patients with IPF compared with healthy lung tissue. Interestingly, this was also seen in fibroblast foci in end-stage sarcoidosis, which, to a large extent, shares gene and protein expression profile with fibroblast foci in IPF.

One potential explanation for the elevated COL7A1 mRNA expression and collagen VII protein deposition in the fibroblast foci may be dysregulated TGF-β signaling, which is the most powerful signaling pathway regulating ECM production and tissue remodeling in lung fibrosis.^42^^,^^43^ In this study, COL7A1 mRNA was found to be up-regulated in distal control lung fibroblasts following the TGF-β1 treatment. Although not statistically significant in TGF-β1–treated IPF fibroblasts, there was a clear trend toward COL7A1 mRNA up-regulation. In a study by Merl-Pham et al,^44^ a proteomic analysis of ECM proteins deposited by IPF fibroblasts cultured for 48 hours, with or without TGF-β1 stimulation, was conducted. Supporting the observations in the present study, the authors also found collagen VII expression in IPF fibroblasts. While unstimulated fibroblasts produced low levels of collagen VII, TGF-β1 stimulation resulted in the highest mean fold change in collagen VII compared with other collagen types. Interestingly, COL7A1 mRNA expression has also been demonstrated to be triggered by TGF-β1 in primary ATII cells, as well as by a fibrosis cocktail (containing TGF-β1 and other IPF-relevant cytokines) in ATII cells derived from human induced pluripotent stem cells.^45^^,^^46^

The main finding of this study was that the striking increase in basal cell populations within the pathologic airways and honeycomb structures in the IPF lung was accompanied by enhanced collagen VII protein deposition in their basement membrane zone. Furthermore, using RNA *in situ* hybridization and public RNA-sequencing data, the classical KRT5^+^ basal cells and KRT5^–^/KRT17^+^ aberrant basaloid cells were corroborated to be the main cell types responsible for COL7A1 mRNA expression. Increasing evidence indicates an important involvement of airway and abnormal epithelial cells in the pathology of IPF.^47^ In line with these observations, several studies have reported alterations of airway morphology, such as dilation of proximal and distal airways, thickening of the airway wall, and abnormal proliferation and differentiation of various epithelial cells.^5^^,^^48^^,^^49^ Additionally, a reduction in terminal bronchioles in fibrotic lungs correlates with an increased number of honeycomb cysts. Recent single-cell RNA-sequencing studies further support these observations, showing a considerable reduction in alveolar epithelial cells and an increase in airway epithelial cells in IPF lungs.^7^^,^^8^ These studies highlighted the newly identified aberrant basaloid cells, a population defined by KRT5^–^/KRT17^+^ expression, which appears to be specific for pulmonary fibrosis and does not exist in normal lung tissue. These cells also express other basal cell markers such as p63, LAMB3, and LAMC2, as well as markers of epithelial-mesenchymal transition (eg, COL1A1, VIM, TNC, FN1, CDH2), and senescence (eg, CDKN1A, CDKN2A, and GDF15), in addition to MMP7, a well-established biomarker in IPF.^7^^,^^8^^,^^50^ In IPF tissue, the aberrant basaloid cells have been described as localizing to the epithelium overlying fibroblast foci.^7^^,^^8^ Epithelial cells covering fibroblast foci were identified (Figure 6, F and G, and Supplemental Figure S9), corresponding to the reported location of aberrant basaloid cells from RNA-sequencing studies. However, in contrast, these cells were not negative for KRT5 at the protein level. Instead, KRT5^–/^KRT17^+^ cells were found in small clusters in preserved alveolar regions with fibrotic septa in two patients with IPF. Although these cells were not surrounded by collagen VII, some displayed intracellular collagen VII. In several fibroblast foci from all examined IPF blocks, KRT17^+^/KRT5^+^ cells containing intracellular collagen VII were found in the intermediate epithelial layer, surrounded by negative luminal epithelial cells above and pathologic fibroblasts below, often with an impaired integrity of collagen VII in the basement membrane zone underneath (Figure 6, G–I, and Supplemental Figure S9). This population of cells may correspond to squamous metaplastic cells previously identified in sandwich fibroblast foci, which co-express intracellular LAMC2, KRT5, p63, and mesenchymal markers vimentin and fibronectin.^51^^,^^52^ Furthermore, they partially lose E-cadherin expression and display positivity for fascin and heat shock protein-27, proteins involved in cell motility. The re-analyzed RNA-sequencing data in this study also showed that both basal cells and aberrant basaloid cells, along with COL7A1, had up-regulated mRNA for anchoring proteins and hemidesmosomes, suggesting an active re-epithelialization process. This process is well described in skin as one of the wound healing phases, which involves keratinocyte migration, proliferation, differentiation, and anchoring.^53^ A study from Nyström et al^54^ in skin indicated that collagen VII is crucial for the re-epithelialization of wounds, orchestrating laminin-332/integrin α6β4 signaling needed for epithelial cell migration, in addition to supporting fibroblast migration and cytokine production in the granulation tissue. This implies that collagen VII may have more functions in the lung beyond mechanical stabilization of the epithelium in the remodeled airways and honeycomb cysts, but further functional studies are needed to explore this. Interestingly, there is also emerging evidence that ATII cells can transdifferentiate into basal-like cells in fibrotic tissues and *in vitro* models.^45^^,^^55^^,^^56^ On the basis of radiology, honeycombing is considered to represent the late stage of IPF; however, due to the unavailability of lung tissue from earlier stages of IPF, little is known about the molecular and cellular changes preceding on the microscopic level. Whether the aberrant re-epithelialization is driven by migration of basal cells from the airways, by transdifferentiation of ATII cells, or both, and how collagen VII, together with other components of the basement membrane niche, contribute to this phenomenon remains to be elucidated.

One limitation of this study is the small cohort of patients included, as access to IPF human lung material is limited. The advantage of end-stage explanted lung tissue used in this study is that it provides a large amount of tissue to be examined and offers valuable insight into the diversity of pathologic changes. Such changes can be missed in small lung biopsies that additionally often present a health risk to severely ill patients. A major strength of this study was the use of human material itself. Although animal models of IPF are commonly used, they often do not replicate all aspects of the human IPF. Until recently, honeycombing could not be mimicked in the bleomycin model.^50^^,^^57^ This limitation could make the study of collagen VII difficult.

In conclusion, collagen VII, an under-investigated ECM protein in IPF, was elevated in the distal lungs of patients with IPF compared with control lungs. In IPF, collagen VII was primarily localized to re-epithelialized areas with aberrant airways and honeycombing, as well as to fibroblast foci—histopathologic features of UIP in IPF (Figure 7). Both epithelial and fibroblast cell populations expressed COL7A1 mRNA, which was also up-regulated in TGF-β1–stimulated fibroblasts, suggesting a role in IPF pathogenesis. As basement membranes are critical for epithelial cell fate determination, further investigation of the role of collagen VII and other related proteins from the basement membrane zone can aid in a better understanding of the complex cell-ECM interactions that lead to the aberrant re-epithelialization of the fibrotic lung.

## Disclosure Statement

None declared.

## Supplemental Data

Supplemental Figure S1 Sankey diagram showing annotations of cell clusters from the data set of Habermann et al^8^ (*https://www.ncbi.nlm.nih.gov/geo*; accession number GSE135893) after reference mapping according to the Human Lung Cell Atlas (HLCA). HAS1, hyaluronan synthase 1; PLIN2, perilipin 2.

Supplemental Figure S2 Controls for polyclonal monospecific anti–collagen VII antibody. Images of positive immunohistochemistry control (skin; **A**) and negative controls omitting the primary anti–collagen VII antibody [skin (**B**) and bronchus (**C**)]. **Arrow** indicates collagen VII in the basement membrane zone. Scale bar = 100 μm (**A**–**C**).

Supplemental Figure S6 Normalized expression level of genes related to anchoring complex and hemidesmosomes in the basement membrane zone in selected epithelial cell populations in control (blue) and idiopathic pulmonary fibrosis (pink) lungs, presented as violin plots. Data derived from the study by Habermann et al^8^ (*https://www.ncbi.nlm.nih.gov/geo*; accession number GSE135893). AT1, alveolar type I cell; AT2, alveolar type II cell; KRT, keratin.

Supplemental Figure S7 Spatial distribution of COL7A1 mRNA in control distal lungs. Images of COL7A1 mRNA in control (**A**) and idiopathic pulmonary fibrosis (IPF; **B**) distal lung tissue detected by *in situ* hybridization. Images of hematoxylin and eosin (H&E) staining (**C**, **F**, and **I**) and COL7A1 mRNA (**D**, **E**, **G**, **H**, **J**, and **K**) on consecutive sections of distal lung tissue of controls, showing COL7A1^+^ epithelial cells (**arrows**) localized to bronchi (**D** and **E**), larger bronchiole (**G** and **H**), and smaller bronchiole (**J** and **K**). Scale bars: 1 mm (**A** and **B**); 100 μm (**C**–**K**).
