# Supplementary figures and images for: Collagen VII Is Associated with Airway Remodeling, Honeycombing, and Fibroblast Foci in Usual Interstitial Pneumonia/Idiopathic Pulmonary Fibrosis
Source: Am J Pathol. 2025 Apr 29;195(8):1467–83. doi: 10.1016/j.ajpath.2025.03.013 (PMC12405904; doi:10.1016/j.ajpath.2025.03.013)

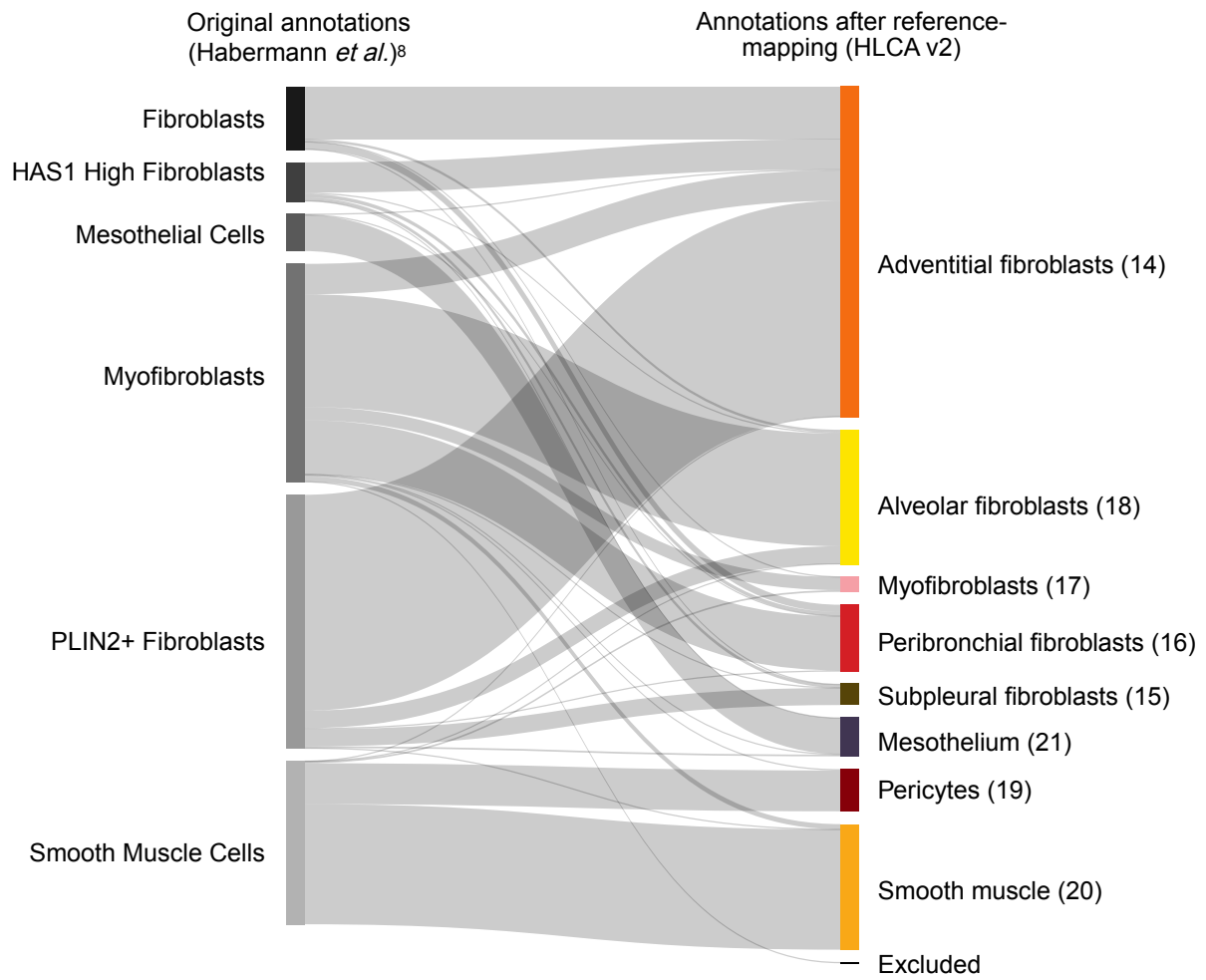

Supplement: Supplemental Figure S1 — Sankey diagram showing annotations of cell clusters from the data set of Habermann et al8 (https://www.ncbi.nlm.nih.gov/geo; accession number GSE135893) after reference mapping according to the Human Lung Cell Atlas (HLCA). HAS1, hyaluronan synthase 1; PLIN2, perilipin 2. [file mmc1.pdf]

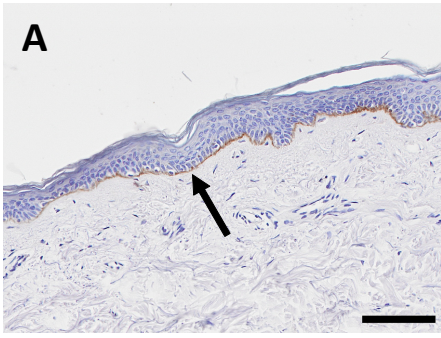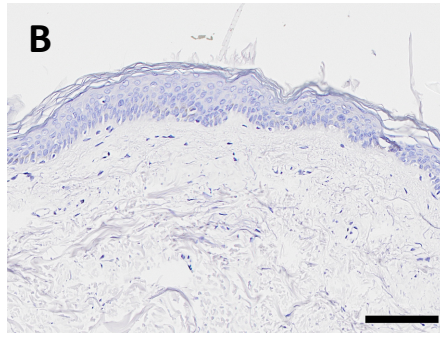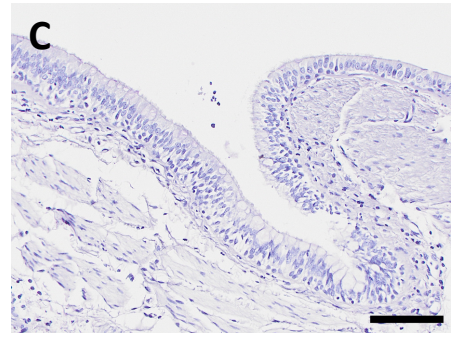

Supplement: Supplemental Figure S2 — Controls for polyclonal monospecific anti–collagen VII antibody. Images of positive immunohistochemistry control (skin; A) and negative controls omitting the primary anti–collagen VII antibody [skin (B) and bronchus (C)]. Arrow indicates collagen VII in the basement membrane zone. Scale bar = 100 μm (A–C). [file mmc2.pdf]

**LAMA3**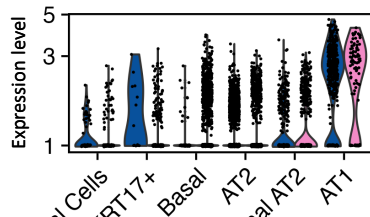**LAMB3**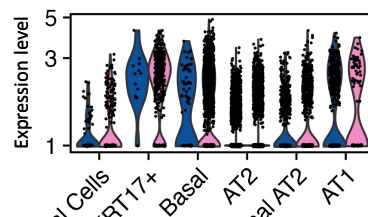**LAMC2**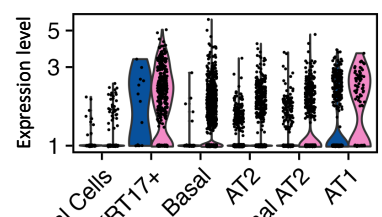**ITGA6**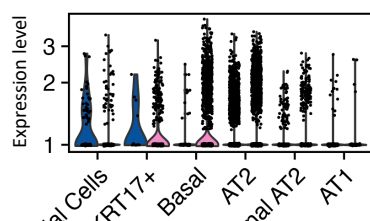**ITGB4**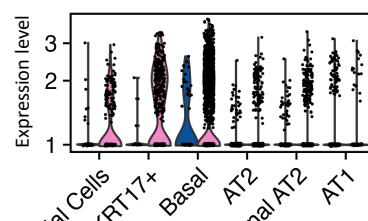**PLEC**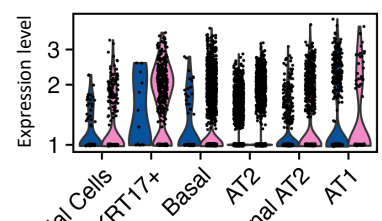**CD151**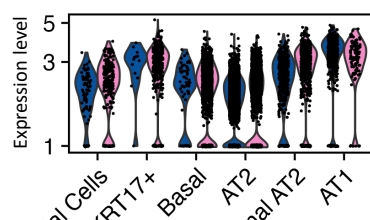**DST**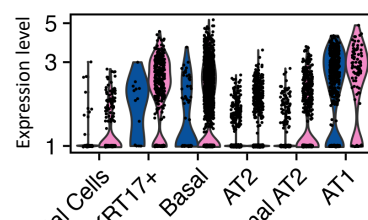**COL17A1**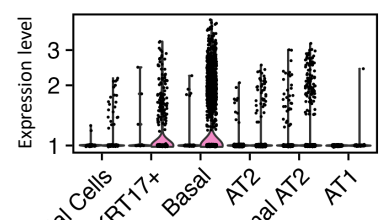

Supplement: Supplemental Figure S6 — Normalized expression level of genes related to anchoring complex and hemidesmosomes in the basement membrane zone in selected epithelial cell populations in control (blue) and idiopathic pulmonary fibrosis (pink) lungs, presented as violin plots. Data derived from the study by Habermann et al8 (https://www.ncbi.nlm.nih.gov/geo; accession number GSE135893). AT1, alveolar type I cell; AT2, alveolar type II cell; KRT, keratin. [file mmc3.pdf]

Control lung

IPF lung

COL7A1

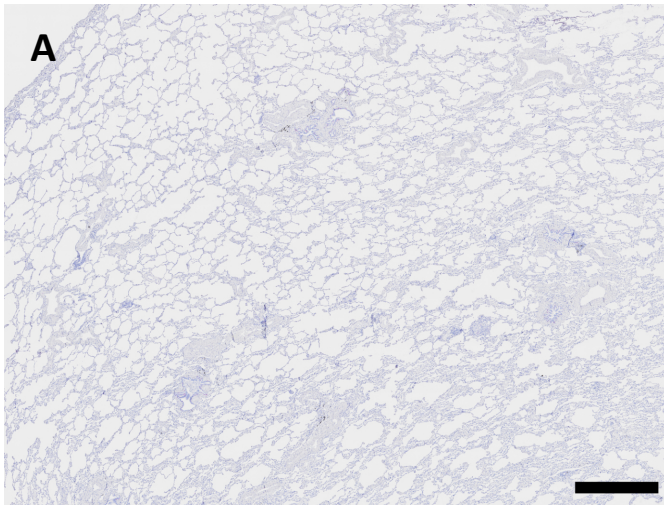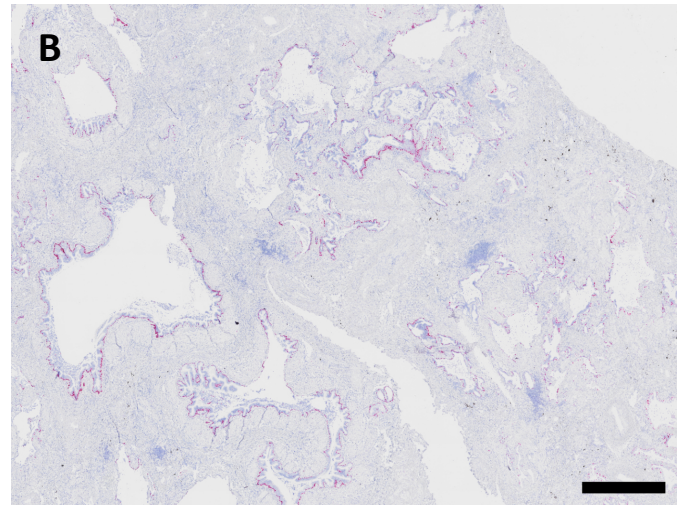

H&E

COL7A1

COL7A1

Control distal lung

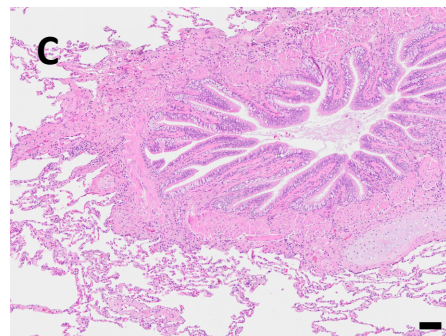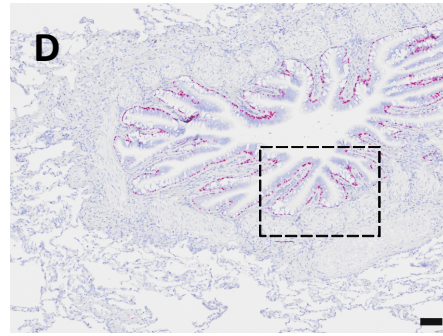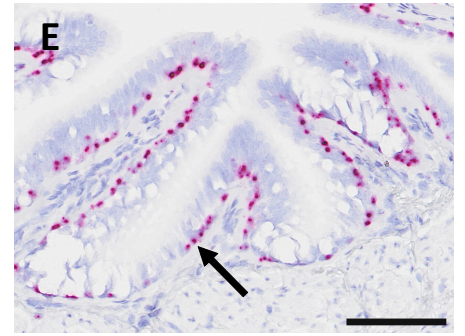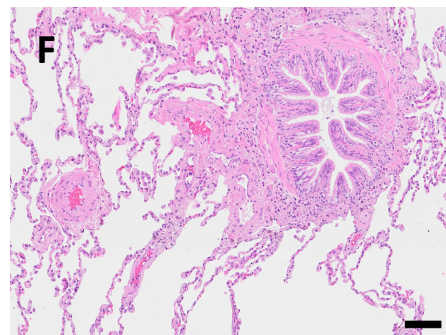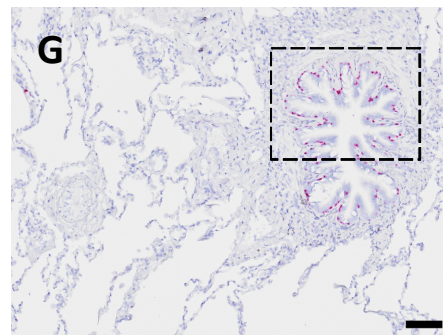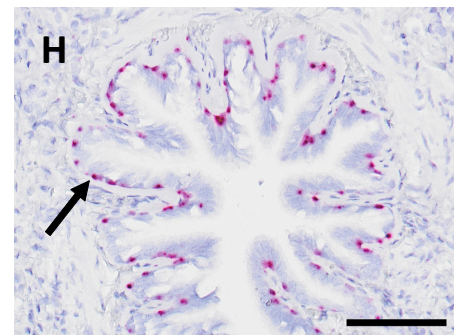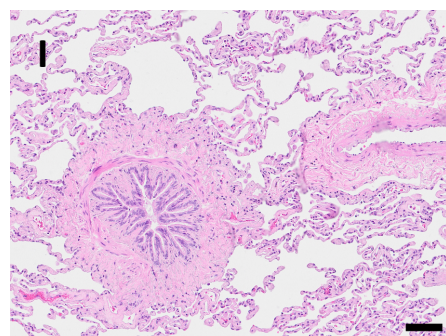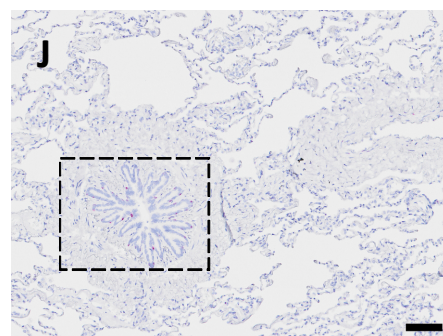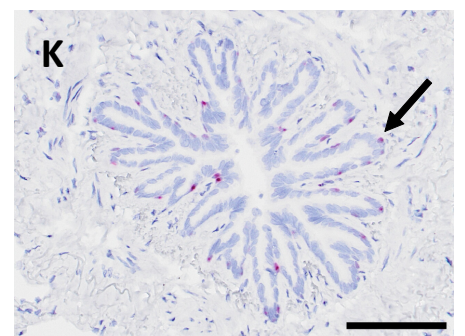

Supplement: Supplemental Figure S7 — Spatial distribution of COL7A1 mRNA in control distal lungs. Images of COL7A1 mRNA in control (A) and idiopathic pulmonary fibrosis (IPF; B) distal lung tissue detected by in situ hybridization. Images of hematoxylin and eosin (H&E) staining (C, F, and I) and COL7A1 mRNA (D, E, G, H, J, and K) on consecutive sections of distal lung tissue of controls, showing COL7A1+ epithelial cells (arrows) localized to bronchi (D and E), larger bronchiole (G and H), and smaller bronchiole (J and K). Scale bars: 1 mm (A and B); 100 μm (C–K). [file mmc4.pdf]
