# Supplementary material for: Collagen VII Is Associated with Airway Remodeling, Honeycombing, and Fibroblast Foci in Usual Interstitial Pneumonia/Idiopathic Pulmonary Fibrosis
Source: Am J Pathol. 2025 Apr 29;195(8):1467–83. doi: 10.1016/j.ajpath.2025.03.013 (PMC12405904; doi:10.1016/j.ajpath.2025.03.013)
Supplement: Supplemental Table S1 [file mmc5.docx]

| Sample | Sex | Age (years) | Smoking status | IHC | ISH | IF | Cell experiments |
| --- | --- | --- | --- | --- | --- | --- | --- |
| CTRL1 | male | 39 | never smoker |  |  |  | ✓ |
| CTRL2 | male | 62 | never smoker | ✓ |  | ✓ | ✓ |
| CTRL3 | male | 62 | former smoker | ✓ | ✓ | ✓ | ✓ |
| CTRL4 | male | 43 | former smoker | ✓ | ✓ | ✓ | ✓ |
| CTRL5 | male | 68 | never smoker | ✓ | ✓ | ✓ |  |
| CTRL6 | male | 66 | current smoker | ✓ |  | ✓ |  |
| CTRL7 | female | 65 | never smoker | ✓ |  | ✓ | ✓ |
| CTRL8 | male | 74 | former smoker |  |  |  | ✓ |
| IPF1 | female | 57 | former smoker | ✓ |  | ✓ | ✓ |
| IPF2 | female | 62 | former smoker |  |  |  | ✓ |
| IPF3 | male | 68 | former smoker | ✓ |  | ✓ | ✓ |
| IPF4 | male | 57 | former smoker | ✓ | ✓ | ✓ | ✓ |
| IPF5 | male | 51 | never smoker |  | ✓ | ✓ |  |
| IPF6 | female | 65 | former smoker | ✓ | ✓ |  |  |
| IPF7 | male | 60 | former smoker | ✓ |  | ✓ |  |
| IPF8 | male | 58 | never smoker | ✓ |  | ✓ |  |

***Supplemental Table S1*** *Details of patient material and its use in experiments.*
